# Characterization of the upper and lower respiratory tract microbiota in Piedmontese calves

**DOI:** 10.1186/s40168-017-0372-5

**Published:** 2017-11-21

**Authors:** Isabella Nicola, Francesco Cerutti, Elena Grego, Iride Bertone, Paola Gianella, Antonio D’Angelo, Simone Peletto, Claudio Bellino

**Affiliations:** 1 Department of Veterinary Sciences, Clinical section, University of Turin, Largo Paolo Braccini 2, 10095 Grugliasco, TO Italy; 2 Istituto Zooprofilattico Sperimentale del Piemonte, Liguria e Valle d’Aosta, Via Bologna 148, 10154 Turin, TO Italy

**Keywords:** 16S rRNA gene, Metabarcoding, Microbiota, Respiratory tract, Bovine respiratory disease

## Abstract

**Background:**

The microbiota of the bovine upper respiratory tract has been recently characterized, but no data for the lower respiratory tract are available. A major health problem in bovine medicine is infectious bronchopneumonia, the most common respiratory syndrome affecting cattle. With this study, we used 16S rRNA gene sequencing to characterize and compare the microbial community composition of the upper and lower respiratory tracts in calves.

**Results:**

The microbiota of the upper (nasal swab [NS]) and the lower (trans-tracheal aspiration [TTA]) respiratory tracts of 19 post-weaned Piedmontese calves with (8/19) and without (11/19) clinical signs of respiratory disease, coming from six different farms, was characterized by 16S rRNA gene metabarcoding. A total of 29 phyla (29 in NS, 21 in TTA) and 305 genera (289 in NS, 182 in TTA) were identified. *Mycoplasma* (60.8%) was the most abundant genus identified in both the NS (27.3%) and TTA (76.7%) samples, followed by *Moraxella* (16.6%) in the NS and *Pasteurella* (7.3%) in the TTA samples. *Pasteurella multocida* (7.3% of total operational taxonomic units [OTUs]) was the most abundant species in the TTA and *Psychrobacter sanguinis* (1.1% of total OTUs) in the NS samples. Statistically significant differences between the NS and the TTA samples were found for both alpha (Shannon index, observed species, Chao1 index, and Simpson index; *P* = 0.001) and beta (Adonis; *P* = 0.001) diversity. Comparison of the NS and TTA samples by farm origin and clinical signs revealed no statistical difference (*P* > 0.05), except for farm origin for the NS samples when compared by the unweighted UniFrac metric (*P* = 0.05).

**Conclusions:**

Using 16S rRNA gene sequencing, we characterized the microbiota of the upper and lower respiratory tracts of calves, both healthy individuals and those with clinical signs of respiratory disease. Our results suggest that environmental factors may influence the composition of the upper airway microbiota in cattle. While the two microbial communities (upper and lower airways) differed in microbial composition, they shared several OTUs, suggesting that the lung microbiota may be a self-sustaining, more homogeneous ecosystem, influenced by the upper respiratory tract microbiota.

**Electronic supplementary material:**

The online version of this article (10.1186/s40168-017-0372-5) contains supplementary material, which is available to authorized users.

## Background

In the last decade, the use of high-throughput sequencing methods (next-generation sequencing, NGS) coupled to DNA barcoding has advanced the study of bacterial communities as a whole [1]. DNA metabarcoding allows the generation of multiple reads of the hypervariable regions of the 16S rRNA gene in a single run, thus yielding a wealth of phylogenetic information in a single experiment [1]. The 16S rRNA gene metabarcoding approach applied to lower respiratory tract samples from humans led to the discovery that the lung is not sterile even in healthy conditions [2]. The finding of lung microbiota in a healthy subject was initially explained as a temporal contamination from the upper respiratory tract either during sampling or by microaspiration [3]. However, recent studies have shown that the microbiota of the lung could be considered an ecosystem and that its composition depends on the immigration, elimination, and reproduction rates of the microbial communities present there [4–6]. Though the components of this ecosystem derive from the upper respiratory tract, they could be able to proliferate in the lungs and form a self-sustaining lung microbiota [4–6]. DNA metabarcoding analysis of pathological respiratory samples led to the hypothesis that alteration of the lung microbiota could assume a key role in the pathogenesis of human lung diseases, including bacterial pneumonia [6, 7].

Study of the bovine respiratory tract holds huge importance because one of the main health issues in the cattle industry is infectious bronchopneumonia, defined as bovine respiratory disease (BRD) [8, 9]. In Europe, BRD is associated with high morbidity and mortality, which leads to a widespread use of antimicrobials and, therefore, increases the alarm about antibiotic resistance [10–19]. BRD is a multifactorial disease resulting from interactions between management, physiologic, and environmental factors and etiological agents [20, 21]. The bacterial pathogens most often identified by culture-based methods in the course of BRD are *Mannheimia haemolytica*, *Pasteurella multocida*, *Histophilus somni*, and *Mycoplasma bovis* [22]. Considered ubiquitous inhabitants of the bovine upper respiratory tract, these bacterial species can proliferate in the lungs when inhaled during stressful events or viral infections [22]. However, they have also been isolated from the lower respiratory tract of calves not presenting clinical signs, raising questions about their role in the development of BRD [23, 24]. A better understanding of the etiology of BRD is critical to improve animal health.

In cattle, 16S rRNA gene metabarcoding to characterize the microbiota of the upper respiratory tract has been applied on nasal swab samples from dairy and beef calves [25–31]. Timsit et al. [26] and Holman et al. [28], for example, reported that the upper respiratory tract microbiota of beef cattle is not stable in the first 40 days on feedlot, possibly explaining their higher susceptibility for developing BRD in this period. Moreover, Lima et al. [31] found that the upper respiratory tract microbiota of dairy calves significantly differed depending on the animal’s clinical respiratory status. The application of this new technique to respiratory tract samples has attracted increasing interest; however, to our knowledge, data about the bovine lower respiratory tract microbiota are still lacking. The goal of this study was to characterize the microbiota of the upper and lower respiratory tracts by applying 16S rRNA gene metabarcoding on nasal swab (NS) and trans-tracheal aspiration (TTA) samples from post-weaned Piedmontese calves with and without clinical signs of BRD.

## Methods

### Sample population and sample collection

The study was performed in Piedmont, northwest Italy. The six farms included in the study were located in the provinces of Torino (TO), Cuneo (CN), and Vercelli (VC). The median distance as the crow flies between the farms was 50 km (min-max, 11–97 km) (map given in Additional file 1). The six farms were located in a restricted area with similar geographical and climatic characteristics. The farms were all cow-calf operations with the same animal management. Briefly, the calves were held with the mother until the end of the weaning period (5 months), then moved to multiple straw-bedded boxes (5–10 animals) with free access to water and feed (roughage and concentrate).

The animals included were post-weaned Piedmontese calves receiving veterinary care by the Veterinary Teaching Hospital of the University of Turin. Medical history and physical examination data were collected via a standardized collection form for each calf. A complete physical examination was performed for all animals focusing on typical clinical signs of BRD: cough, nasal discharge, and abnormal sounds at thoracic auscultation (wheezes, crackles) (Master Classic II™, 3M Littmann® stethoscope, 3M, St. Paul, MN, USA). Calves that had been treated in the week prior to examination or presented clinical signs suggestive of diseases different from BRD were excluded from the study.

Nasal swab (NS) samples were collected using sterile swabs (17 cm, DrySwab, Copan S.p.A, Italy) from both nostrils of each calf after cleaning the nostrils with 90% ethyl alcohol. Trans-tracheal aspiration (TTA) was performed as previously described [23]. Briefly, the animals were mildly sedated with intravenous injection of 0.05 mg/kg Xylasine (Rompun®, Bayer Healthcare, Germany), and an area of 3 × 3 cm about 7–10 cm caudal to the larynx was shaved and surgically prepared with 90% ethyl alcohol and iodophors. The area was desensitized with 4% procaine hydrochloride (Aticain®, A.T.I., Italy), and a longitudinal 1-cm incision was then placed in the midline directly above the trachea. A 12-G needle was used to perforate the trachea between two cartilage rings. A male dog urinary catheter (2 mm × 50 cm; Buster, sterile dog catheter, Kruuse, Germany) was introduced into the needle and pushed down into the airway for about 45 cm. Finally, a volume of 50 ml sterile 0.9% saline solution was injected through the catheter and immediately aspirated. To prevent sample contamination, a new sterile kit and a new pair of sterile gloves were used for each calf. All samples were stored at − 80 °C until analysis.

### DNA extraction and library preparation

#### DNA extraction

DNA was isolated using DNAzol® reagent (Invitrogen, Carlsbad, CA, USA) according to the manufacturer’s instructions. Briefly, the NS were dipped in 500 μl of DNAzol® reagent immediately after collection, while the TTA fluids were pelleted and added with 1 ml of DNAzol® reagent, after thawing at 4 °C. The samples were then repeatedly homogenized and incubated at 4 °C for 18 h. After DNA precipitation by means of ethanol, the pellets were rinsed twice. Finally, DNA was eluted in 30 μl of RNase and DNase free water. The DNA concentration of each sample was determined using a Qubit fluorimeter (Qubit®, Invitrogen) and normalized to 5 ng/μl. Samples with a lower DNA concentration were not processed further.

#### Library preparation

The normalized DNA was processed according to the 16S Metagenomic Sequencing Library Preparation protocol as recommended by Illumina (Illumina, San Diego, CA, USA). Briefly, 12.5 ng of genomic DNA underwent an initial PCR step with the 16S amplicon PCR forward and reverse primers targeting the V3 and V4 regions of the 16S rRNA gene [32], followed by PCR cleanup with Agencourt Ampure XP (Beckman Coulter, Brea, CA, USA) magnetic beads, and index PCR for a second cleanup with magnetic beads. Normalization was based on the average size of the library as assessed with an Agilent High Sensitivity DNA Kit on a 2100 Bioanalyzer (Agilent Technologies, Santa Clara, CA, USA) and quantification with a NEBNext® Library Quant Kit for Illumina® (New England Biolabs, Ipswich, MA, USA) normalization. The normalized libraries were eventually pooled and loaded for sequencing on an Illumina MiSeq platform with paired-end 2 × 300 bp protocol using a MiSeq® Reagent Kit ver. 3 (600 cycles) (Illumina).

Neither blank controls nor mock communities were included in the present study; however, in order to limit the influence of contamination by extraction and amplification on the analysis, all samples were processed using the same DNA extraction reagents, and the amplifications were conducted with the same reagent lots.

### Bioinformatic and statistical analysis

Reads were processed for quality filtering (using Q30 as threshold), adapter and primer removal BBDuk2 ver. 36.14; mate pairing was performed with BBMerge ver. 9.00 (https://sourceforge.net/projects/bbmap/). The fasta files were then processed using a Quantitative Insights Into Microbial Ecology (QIIME) 1.9.1 pipeline [33]. In detail, paired reads were merged in a single fasta file with *multiple_split_libraries_fastq.py* script, which provides further quality filtering. Operational taxonomic unit (OTU) picking was performed with the *pick_open_reference_otus.py* using the UCLUST method and with 97% identity to the Greengenes (version gg_13_8) reference database, followed by de novo clustering [34]. Representative sequences were checked for de novo chimera detection using ChimeraSlayer integrated in QIIME, and the chimeras were then filtered out from the OTU table [35]. The alpha and beta diversities were computed with the *core_diversity_analyses.py* script, rarefying the samples at 2500 reads. Samples with less than 2500 reads were excluded from the statistical analysis because these were not representative. Phyla, genera, and species abundance was reported as overall relative abundance, average relative abundance, and standard error of the mean (SEM).

Statistical support to the alpha diversity comparison between groups was assessed by nonparametric test with Monte Carlo permutations implemented in the *compare_alpha_diversity.py* script, while for the variance within and between groups, QIIME wrapper *compare_categories.py* was applied with permutational multivariate analysis of variance (PERMANOVA, Adonis method from *R* package Vegan implemented in QIIME). Statistically significant differences in OTU frequencies based on non-normalized raw counts between the NS and TTA samples from clinically healthy animals and those presenting at least one clinical sign suggestive of BRD were assessed using the *differential_abundance.py* script that implements the *R* package DESeq2 [36], and *P* values were adjusted (*P*adj) for multiple-testing with the false discovery rate (FDR) procedure of Benjamini and Hochberg [37]. OTUs were considered differentially abundant if at *P*adj ≤ 0.05 and if the estimated fold change was > 1.5 or < 1/1.5.

In order to evaluate the type I and type II errors and strengthen the results, we performed a power analysis on the data grouped by sample type, and by sample type and clinical signs according to the method presented by La Rosa and colleagues, implemented in the R package *HMP*, using the *MC.Xmcupo.statistics* function and 1000 Monte Carlo experiments [38].

## Results

### Study population

The number of calves selected from each farm ranged from 1 to 7, for a total of 22 calves (17 males and 5 females), aged from 5 to 14 months. Thirteen out of 22 animals (59%) were clinically healthy, and the remaining nine showed at least one clinical sign attributable to BRD (Table 1). Nasal swab samples were collected from 17 out of 22 animals, and TTA samples were collected from all animals. DNA was successfully extracted at a concentration above 5 ng/μl from 32 samples (13 NS and 19 TTA fluid samples). In one of the nine animals presenting clinical signs, the DNA concentration was below 5 ng/μl in both the NS and TTA samples; this animal was excluded from the study. At least one sample (NS or TTA) from 21 out of 22 calves was available for analysis. Further details regarding the study population are reported in Table 1.

**Table 1:** Data about calves (origin, signalment, history, clinical examination) and number of reads of each sample

ID	Farm	Age (months)	Sex	Weight (kg)	Previous episodes of BRD	Spontaneous cough	Nasal discharge	Abnormal lung sounds	Read number NS sample	Read number TTA sample
1^a^	TO1	14	M	400	3 months before sampling	Repeated	Cloudy and bilateral	Wheezes and crackles on the left side of the thorax	NP	53,494
2^b^	CN1	6	M	180	No	Absent	Absent	Absent	NP	7902
3^a^	CN1	6	M	180	1 month before sampling	Occasional	Absent	Wheezes and crackles on both sides of the thorax	NP	47,115
4^b^	CN1	6	M	180	No	Absent	Absent	Absent	NP	18,855
5^b^	CN1	6	M	180	No	Absent	Absent	Absent	NP	81,564
6^a^	CN2	6	M	180	No	Absent	Cloudy and unilateral	Absent	36,160	129,750
7^b^	CN2	6	M	180	No	Absent	Absent	Absent	28,543	118,454
8^b^	CN2	6	M	180	No	Absent	Absent	Absent	31,232	61,778
9^a^	CN2	6	M	180	No	Absent	Cloudy and bilateral	Wheezes and crackles on both sides of the thorax	DNA < 5 ng/μl	DNA < 5 ng/μl
10^a^	VC1	5	F	150	No	Repeated	Absent	Wheezes and crackles on both sides of the thorax	40,694	142,459
11^a^	VC1	5	F	150	2 months before sampling	Absent	Absent	Wheezes and crackles on both sides of the thorax	12,952	DNA < 5 ng/μl
12^b^	VC1	5	F	150	No	Absent	Absent	Absent	5988	DNA < 5 ng/μl
13^a^	VC1	5	M	150	No	Absent	Absent	Wheezes and crackles on both sides of the thorax	24,070	26,910
14^b^	TO2	6	M	200	No	Absent	Absent	Absent	DNA < 5 ng/μl	68,954
15^b^	TO2	6	F	200	No	Absent	Absent	Absent	20,464	40,790
16^a^	TO2	6	F	200	No	Absent	Absent	Wheezes and crackles on the right side of the thorax	167,198	107,679
17^a^	TO2	6	F	200	No	Absent	Cloudy and bilateral	Absent	DNA < 5 ng/μl	143,886
18^b^	TO2	6	M	200	No	Absent	Absent	Absent	3932	55,630
19^b^	TO2	6	M	200	No	Absent	Absent	Absent	DNA < 5 ng/μl	1,825^c^
20^b^	TO2	6	M	200	No	Absent	Absent	Absent	154,004	4585
21^b^	TO3	5	M	170	No	Absent	Absent	Absent	1,536^c^	6833
22^b^	TO3	5	M	170	No	Absent	Absent	Absent	159^c^	189^c^

ID = animal’s identification number. Farm origin = farms are indicated with the provincial code (*TO* Torino, *CN* Cuneo, *VC* Vercelli) and progressively numerated. Sex: *M* = male, *F* = female. Read number = number of reads for each sample. TTA = trans-tracheal aspiration sample. NS = nasal swab sample. NP = not performed. DNA < 5 ng/μ = samples with DNA concentration lower than 5 ng/μl and not sequenced

^a^Calves with clinical signs of BRD

^b^Healthy calves

^c^Samples with less than 2500 reads excluded from the final analysis

### Genetic analysis

After primer and quality trimming and pair merging, the total read count was 3,482,819, with an average read length of 407 ± 80 bp. After application of the *multiple_split_libraries_fastq.py* script to merge all the sequences in a single file, with further quality checks, the read number was 2,813,497. The total reads classified in the OTU table was 1,645,584, divided in 526,932 from the NS samples (median 24,072, min-max 159–167,198) and 1,118,652 from the TTA fluid samples (median 53,494, min-max 189–143,886). The number of assigned reads was below 2500 in 4 (2 NS and 2 TTA) out of 32 samples, and they were excluded from the analysis. Therefore, the final analysis was performed on 28 samples (11 NS and 17 TTA) from 19 calves (8 with clinical signs, 11 clinically healthy). Of these 28 samples, 18 (64.3%) were matched samples from the same animal (9 NS and 9 TTA) (Table 1). Finally, the reads obtained from these 28 samples were classified in 4368 OTUs (median 226.5, min-max 44–2502). The median (min-max) of the OTUs was 957 (495–2502) in the NS samples and 139 (44–719) in the TTA samples. A total of 810 unique sequences were classified as chimeric sequences and therefore removed from the OTU table before analysis.

### Phylum composition

**Fig. 1:**
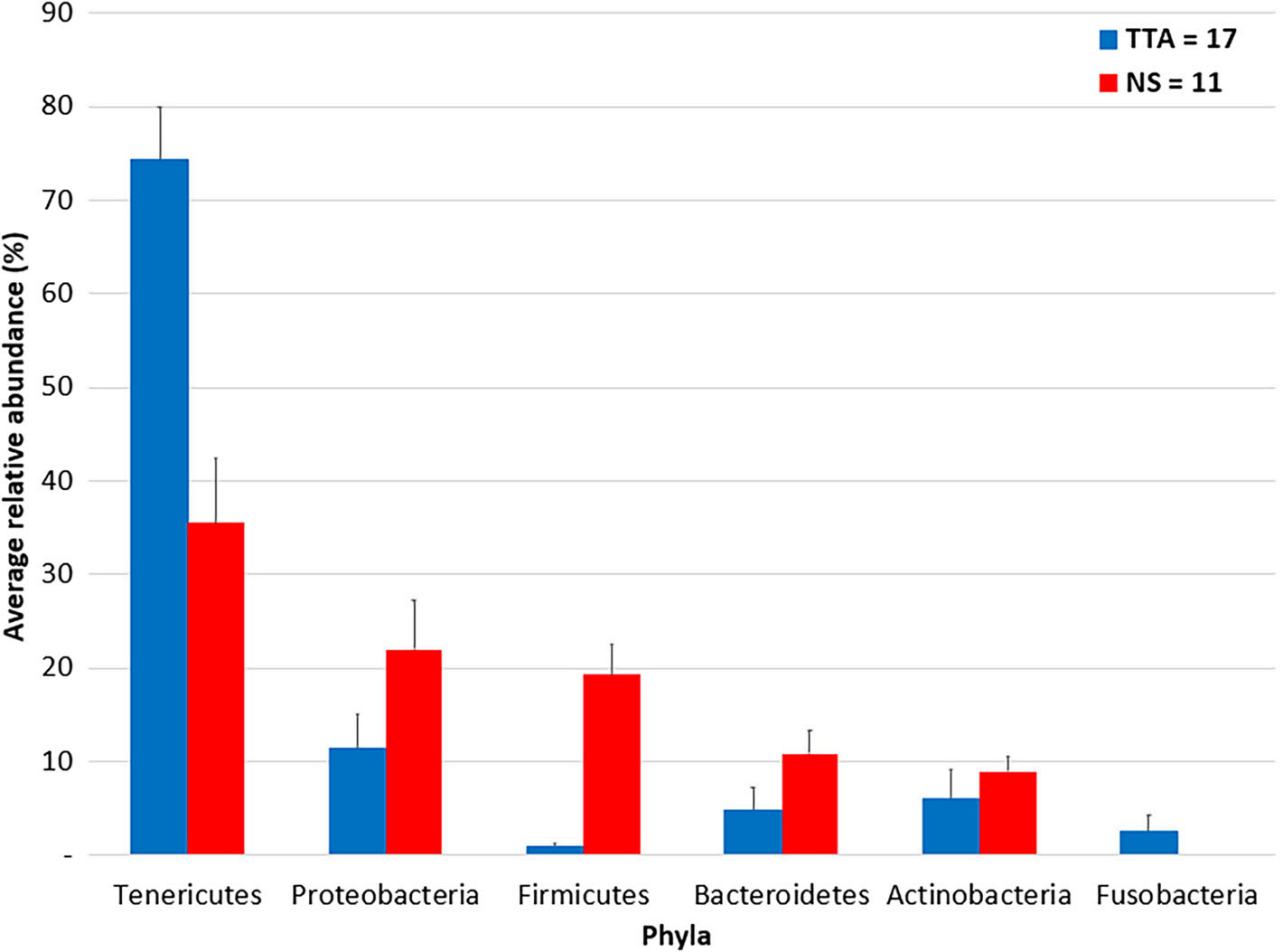
Average relative abundance of phyla in the nasal swab (NS) and trans-tracheal aspiration (TTA) samples. Only phyla with a relative abundance higher than 1% in at least one sample type were represented. Blue columns represent NS (*n* = 11) samples, while red columns represent TTA (*n* = 17). The bars represent the standard error of the mean

Overall, 29 phyla were identified. The microbial community of the samples was structured as follows: Tenericutes (61.8%, 59% ± 5.6%), Proteobacteria (19%, 15.6% ± 3.2%), Firmicutes (6.5%, 8.1% ± 2.2%), Bacteroidetes (5.6%, 7.2% ± 1.8%), Actinobacteria (3.8%, 7.1% ± 2%), Fusobacteria (2.8%, 1.6% ± 1.1%), others (0.3%, 0.4% ± 0.1%), and unassigned (0.2%, 1.1% ± 0.9%). Of the 29 phyla identified in the NS samples, the most abundant were Proteobacteria (36.1%, 21.9% ± 5.3%), Tenericutes (27.7%, 35.4% ± 6.9%), Firmicutes (18.4%, 19.3% ± 3.3%), Bacteroidetes (10.1%, 10.8% ± 2.5%), and Actinobacteria (6.3%, 8.9% ± 1.6%). Only 21 out of 29 phyla were identified in the TTA fluid samples, and the most abundant were Tenericutes (77.9%, 74.3% ± 5.6%), Proteobacteria (11.0%, 11.4% ± 3.7%), Fusobacteria (4.2%, 2.5% ± 1.7%), Bacteroidetes (3.5%, 4.8% ± 2.4%), and Actinobacteria (2.6%, 5.9% ± 3.2%). Figure 1 presents the phylum abundance and composition in the upper and lower respiratory tracts.

### Genera and species composition

**Fig. 2:**
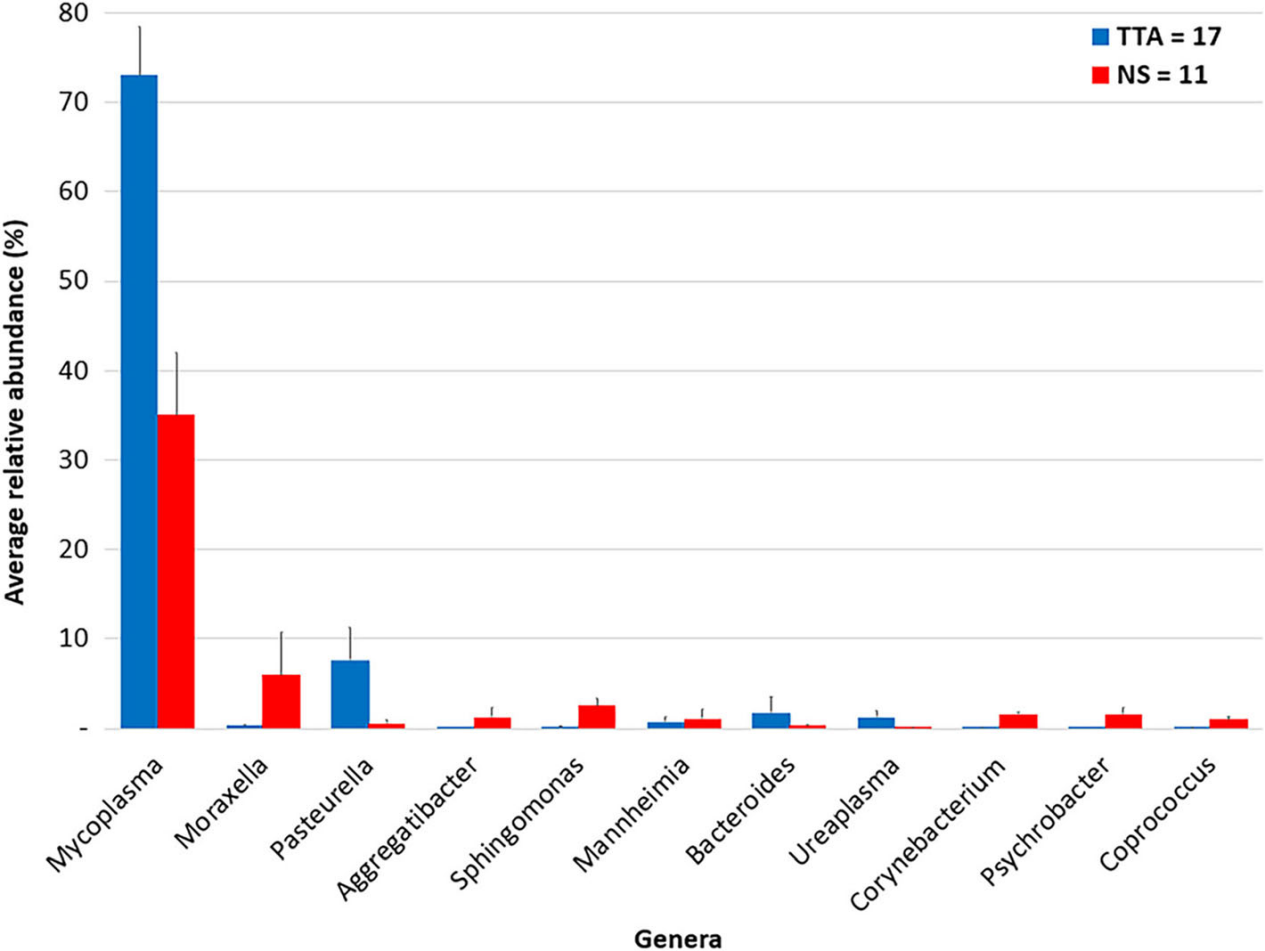
Average relative abundance of genera in the nasal swab (NS) and trans-tracheal aspiration (TTA) samples. Only bacterial genera with a relative abundance higher than 1% in at least one sample type were represented. Blue columns represent NS (*n* = 11) samples, while red columns represent TTA (*n* = 17). The bars represent the standard error of the mean

A total of 305 genera were identified: 289 in the NS samples and 182 in the TTA samples. Overall, the most abundant genera were *Mycoplasma* (60.7%, 58.1% ± 8.9%), *Moraxella* (5.5%, 2.6% ± 3%), and *Pasteurella* (5.2%, 4.9% ± 3.7%). Four other genera with a relative abundance higher than 1% were identified: *Sphingomonas* (1.2%, 1.1% ± 0.6%), *Mannheimia* (1.2%, 0.9% ± 0.9%), *Aggregatibacter* (1.2%, 0.5% ± 0.6%), and *Bacteroides* (1.1%, 1.2% ± 1.7%). *Mycoplasma* was the most abundant genus in both the NS and the TTA fluid samples, with a relative abundance of 27.2% (35.1% ± 6.9%) and 76.5% (72.9% ± 5.5%), respectively, followed by *Moraxella* (16.6%, 5.9% ± 4.8%) in the NS samples and by *Pasteurella* (7.3%, 7.6% ± 3.7%) in the TTA samples. A few other genera with an abundance > 1% were also found in the NS samples [*Aggregatibacter* 3.6% (1.2% ± 1%), *Sphingomonas* 3.4% (2.5% ± 0.8%), *Corynebacterium* 1.3% (1.6% ± 0.2%), *Psychrobacter* 1.2% (1.6% ± 0.6%), *Coprococcus* 1% (1% ± 0.2%)] and the TTA samples [*Mannheimia* 1.6% (0.7% ± 0.6%), *Bacteroides* 1.5% (1.8% ± 1.8%), *Ureaplasma* 1.3% (1.2% ± 0.6%)] (Fig. 2).

Besides *Mycoplasma*, present in all samples, *Delftia*, *Sphingomonas*, and *Agrobacterium* composed the core biota of 90% of the samples. Twelve OTUs were present in all NS samples, including seven orders (*Aeromonadales*, *Actinomycetales*, *Clostridiales*, *Flavobacteriales*, *Mycoplasmatales*, *Saprospirales*, and *Sphingomonadales*) and four genera (*Succinivibrio*, *Mycoplasma*, *Sphingomonas*, and *Corynebacterium*). Overall, 123 out of 305 genera were found only in the NS samples and 16 genera only in the TTA samples (Additional file 2: Table S1).

Considering only the OTUs assigned at the species level, *Pasteurella multocida* (7.3% of total OTUs, 7.6% ± 3.7%) was the most abundant in the TTA fluid samples and *Psychrobacter sanguinis* (1.1% of total OTUs, 1.5% ± 0.6%) in the NS samples. The complete list of all species identified, when possible, is reported in Additional file 2: Table S2.

### Comparison of bacterial composition between TTA fluid and NS samples

Alpha diversity values are reported in Table 2. Good’s coverage estimate with a rarefaction at 2500 was 91.6% ± 2.9% for the NS and 99% ± 0.9% for the TTA fluid samples. The alpha diversity indices and rarefaction curves of each sample are reported in Additional file 3.

**Table 2:** Alpha diversity indexes calculated for the nasal swab (NS) and trans-tracheal aspiration (TTA) samples

	TTA	NS	*P* value
Chao1 index	95.62 ± 78.48	720.74 ± 225.22	0.001
Observed species	40.57 ± 35.21	395.30 ± 152.62	0.001
Shannon index	1.46 ± 0.83	5.14 ± 1.74	0.001
Simpson index	0.45 ± 0.24	0.82 ± 0.13	0.001

Chao1 index, observed species, Shannon’s diversity index, and Simpson index values are reported as mean ± standard error

**Fig. 3:**
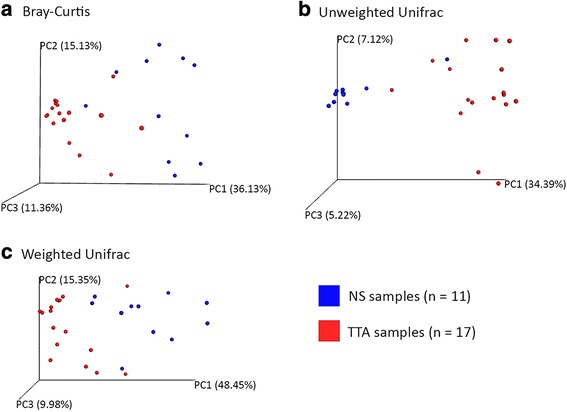
Principal coordinates analysis (PCoA) 3D images. PCoA was performed using Bray-Curtis dissimilarity (**a**), unweighted UniFrac (**b**), and weighted UniFrac (**c**) distance matrices. Each sample is represented by a point with nasal swabs (NS = 11) in blue and trans-tracheal aspiration (TTA = 17) in red. The clustering observed between the NS and TTA samples indicates differences in the microbial compositions of these sampling sites

There was a statistically significant difference in Shannon’s diversity index between the TTA fluids and the NS samples, considering the confidence interval. The microbial composition of the upper and lower respiratory tracts was compared by Bray-Curtis dissimilarity, weighted UniFrac, and unweighted UniFrac phylogenetic distances. The difference between the two bacterial communities was statistically significant as assessed by Adonis (*P* = 0.001), based on the three different distance matrices. Principal coordinates analysis (PCoA) plots of the methods are shown in Fig. 3. The type I error with a significance at 0.05 was < 0.001, as was the type II error, providing a power > 90%.

There were 741 OTUs with a statistically significant difference in abundance (as assessed by DESeq2 analysis) (details given in Additional file 4), with 19 more abundant in the TTA microbiota and 722 in the NS one.

### Correlation of microbiota composition of NS and TTA fluid samples in relation to clinical status and farm of origin

Comparison of the microbiota composition of the TTA samples by farm of origin showed no statistically significant difference for this factor (Adonis on either Bray-Curtis dissimilarity, weighted UniFrac, or unweighted UniFrac distance; *P* > 0.05). It was not possible to estimate the type I and II errors because one group had size = 1. Similarly, no statistical difference in the microbiota composition of the NS samples was found when two statistical methods (Adonis on Bray-Curtis dissimilarity and weighted UniFrac distances; *P* > 0.05) were applied, except when compared based on unweighted UniFrac distance (*P* = 0.05). The type I error with a significance at 0.05 was < 0.001, as well as the type II error, providing a power > 90%. Finally, no correlation was found between the presence or absence of clinical signs and microbial composition of the NS and the TTA samples (Adonis on Bray-Curtis dissimilarity, weighted UniFrac, or unweighted UniFrac distance; *P* > 0.05, power > 90%).

## Discussion

To the best of our knowledge, this is the first study using the 16S rRNA gene metabarcoding approach to characterize and compare the microbiota of the upper and lower respiratory tracts in calves with and without clinical signs of BRD. Recent studies have demonstrated the presence of several bacterial communities in the bovine nasopharynx, documenting that potential pathogenic bacteria are common inhabitants of the upper respiratory tract in cattle [25–31]. Our results are in agreement with the literature as regards the high number of phyla identified and the fact that five phyla seem to be dominant in the bovine upper respiratory tract microbiota: Proteobacteria, Tenericutes, Firmicutes, Actinobacteria, and Bacteroidetes [25, 26, 28–31]*.*

Moreover, we found that the microbiota of the upper respiratory tract differs depending on the animals’ farm of origin. However, the number of calves was not equally distributed among the farms, and this difference was statistically significant based only on the unweighted UniFrac distance, which accounts for the presence/absence of observed OTUs. While it is difficult to draw general conclusions from the present data and further studies are needed to confirm this difference, it is reasonable to assume that environmental factors can influence, at least partially, nasal microbiota composition.

In fact, a variation in microbiota composition of the upper respiratory tract of calves when they are moved in a new environment has already been documented [26, 28]. Nevertheless, several factors have been observed to influence the development and composition of nasopharyngeal microbiota in humans (e.g., mode of birth delivery or breastfeeding) and possibly predispose to the development of respiratory disease [39, 40]. Moreover, differences in microbiota composition in relation to geographical provenience have also been found in humans [41].

A limitation to our study may be the absence of a blank control and a mock community, to calculate the PCR error rate and to evaluate the possible presence of contaminants in the process of DNA treatment. At the time of this study, the inclusion of sequencing controls was not a widespread practice and was not a part of similar studies [25, 26, 28].

Forty-five out of the 305 genera identified were possible contaminants, according to Salter et al. [42], but their relative abundance was low (Additional file 2: Table S1). Four of these genera were found with a relative abundance higher than 1% only in the NS samples: *Sphingomonas*, *Delftia*, *Psychrobacter*, and *Corynebacterium*. The relative abundance of contaminant genera was 0.6% in the TTA and 11.35% in the NS samples. As suggested by Salter et al. [42], since contaminant issues are more relevant in low biomass samples, it could be hypothesized that the bacterial load in the NS samples was lower, which would be odd, given that the upper airways are probably far more exposed to a higher environmental bacterial load [43]. A more probable explanation is that these genera were more abundant in the NS samples because of environmental contamination of the upper respiratory tract, rather than contamination occurring during the samples’ analysis due to low biomass.

Overall, the most abundant genus identified was *Mycoplasma*, which was abundantly found in all NS and TTA samples. This genus was previously identified with high abundance in the upper respiratory airways of both healthy and BRD-affected calves [26, 31]. *Mycoplasma* is spread worldwide, and it has frequently been isolated also with the use of bacterial culture and PCR detection from lower respiratory tract samples from calves, regardless of their clinical status [23, 24]. Unfortunately, in this study, the lack of information about *Mycoplasma* species present in the TTA samples precludes formulating a hypothesis about their pathogenetic role. Although *M. bovis* is recognized as a major etiologic agent in BRD and otitis media, less is known about other *Mycoplasma* spp., e.g., *M. dispar* and *M. bovirhinis*, which have been detected less frequently in the bovine respiratory tract [23, 24, 44–47]. It has been suggested that the lack of isolation of these two species could be correlated with an inhibitory effect of other mycoplasmas [24, 48]. Furthermore, discriminatory analyses within this genus could provide clues to determining the relationship between these species and their role in BRD.

Our observation of *Moraxella*, the second most abundant genus identified in the NS samples, is shared by Timsit et al. [26] and Lima et al. [31]. Lima et al. [31] also found a correlation between the presence of *Moraxella* and the development of BRD. In cattle, *Moraxella bovis* is the etiological agent of infectious bovine keratoconjunctivitis (IBK), and *Moraxella* genus has rarely been isolated in the course of BRD [49, 50]. In children, the role of this genus in the development of respiratory disease is controversial and is probably correlated with the species identified. In fact, it has been associated not only with the development of asthma and respiratory disease but also with a more stable microbiota, which is linked to a lower risk of developing respiratory disease [51–53]. *Corynebacterium* was identified in all NS samples, and previous studies have reported its relatively high abundance in the bovine upper respiratory tract [25, 26, 31]. In children, it has been correlated with a reduced risk of developing otitis media and respiratory disease [39, 54].

Four of the five most abundant phyla identified in the lower respiratory tract were the same as those found in the upper respiratory tract (Proteobacteria, Tenericutes, Actinobacteria, and Bacteroidetes*)*, albeit with different abundance, though Fusobacteria seemed to be characteristic of this ecosystem*.* Identification of this phylum in the bovine upper respiratory tract has rarely been reported and each time with low abundance [25, 30, 31]. In other species, such as the dog and humans, Fusobacteria is one of the most abundant phyla found in the oral cavity but it is less abundant in the nasopharynx [43, 55, 56]. Furthermore, in the human lung, the abundance of Fusobacteria varies in relation to its presence in the oral cavity, leading to the hypothesis that the oral microbial community could be a bacterial source for lung microbiota composition [56–60].

The second most abundant genus identified in the TTA samples, and the main component of the phylum Proteobacteria, was *Pasteurella*. It was comprised mostly of *P. multocida*, and it was also identified with a lower abundance in the NS samples. In contrast, *Pasteurellaceae* were more abundant in oral than in lung and nasal samples from dog, and a higher abundance of *Pasteurellales* were found in the throat of sheep [43, 61]. *P. multocida* is considered a commensal of the upper respiratory airways, but its identification in samples from the lower respiratory tract has usually been correlated with BRD [22, 24]. Nevertheless, Angen et al. [23] isolated *P. multocida* from TTA samples obtained from clinically healthy animals. The absence of a significant difference in lower microbiota composition between healthy and BRD-affected calves suggests that *P. multocida* could be part of the microbial flora of the lower respiratory tract of healthy animals.

Our results showed significant differences in alpha diversity and beta diversity between the upper and the lower respiratory tract microbiota: the number of species composing the lower respiratory tract microbiota was lower, and these species differed by abundance and type from the upper respiratory tract microbiota. The same outcomes were obtained from studies in humans and dogs [3, 5, 60, 62]. In humans, the lung microbiota was found to differ significantly from the oral and the nasal microbiota [5, 62]. In healthy individuals, the lung microbiota partly overlaps with the oral microbiota [5, 62], which is the main source of the composition of the lung microbiota owing to the constant flow of saliva from the mouth, while much less liquid flows from the nose [60]. In upper respiratory tract disease, there is an increased liquid flow from the nose, with a higher potential to affect the lung microbiota as a consequence [60].

Analysis of respiratory tract samples obtained from the dog led to the hypothesis for a self-sustaining lung microbiota also in this species, which proved to be much more homogeneous than the composition of the oral or nasal microbiota [43]. In the present study, the trans-tracheal approach was used for the collection of samples from the lower respiratory tract in order to minimize the contamination from the upper respiratory ways. Moreover, this method has been recommended as optimal for evaluation of the microbiological status of the lower respiratory tract of calves [23]. The finding of 16 genera identified only in the lower respiratory tract, albeit few and with low abundance, suggests that the lung could be colonized by characteristic bacterial species also in cattle. Unfortunately, because of the paucity of information about the oral cavity microbiota of cattle, no conclusions can be drawn.

Comparison by farm of origin showed a statistical difference in microbiota composition only for the NS samples. This suggests that the lower respiratory tract microbiota may be more homogeneous and resilient than the upper respiratory tract, in agreement with the results found in the dog [43]. Moreover, these findings strengthen the hypothesis for a self-sustaining lower respiratory tract microbiota also in cattle.

The absence of significant differences in the bacterial species recovered from the NS and TTA samples from the healthy and the BRD-affected calves contrasts with previous results obtained in humans and cattle [7, 31]. Since BRD is a syndrome and not a single disease with a linear etiology, it may manifest clinically and microbiologically in several different forms [20]. Various other factors could have influenced our results and precluded differentiation between the microbiota of a diseased lung and that of a healthy one. Nevertheless, the primary aim of the present study was to characterize the microbiota of the respiratory airways of calves examined and sampled in field conditions.

## Conclusions

Our findings demonstrate the presence of bacterial communities in the lower respiratory tract in both healthy calves and those presenting clinical signs of BRD. The results obtained from the NS samples suggest that environmental factors (farm, management) may influence the microbial composition of the bovine upper respiratory tract. The finding of bacterial communities in the lower respiratory tract, which differed from the ones found in the upper respiratory tract, suggests the presence of a self-sustaining microbiota at this level. Further studies including characterization of the oral cavity microbiota are needed to confirm this hypothesis.

## Additional files

Additional file 1: Geographical distribution of the farms. Maps were adapted from Google Maps. (PDF 158 kb)

Additional file 2: Table S1. Relative abundance of genera identified in the nasal swab (NS) and the trans-tracheal aspiration (TTA) samples. Genera identified only in the NS (*n* = 11) or in the TTA (*n* = 17) samples are shown in bold or underlined, respectively. **Table S2.** Operational taxonomic units (OTUs) identified at the species level in the nasal swab (NS) and trans-tracheal aspiration (TTA) samples. (DOCX 44 kb)

Additional file 3: Alpha diversity metrics per sample. The alpha diversity metrics include Good’s coverage, Chao1 and observed species indices which estimate species richness, and Simpson and Shannon indices which estimate species evenness. (PDF 827 kb)

Additional file 4: A table reporting the differential abundances of operational taxonomic units (OTUs) found in the nasal swabs (NS = 11) and in the trans-tracheal aspiration (TTA = 17) samples obtained by the DESeq2 analysis. As reported in the DESeq2 support information, the “base mean” column reports the mean of normalized counts for all samples, while the “log2FoldChange” column reports the log fold change calculated for the TTA as compared to the NS samples, with the relative standard error in the adjacent column “lfcSE” (that is log2 fold change standard error). For statistical significance, *P* values are reported without (*P* value) and with adjustment (*P*adj) for multiple testing with the false discovery rate (FDR). (XLS 1114 kb)

## Abbreviations

BRD: Bovine respiratory disease
DNA: Deoxyribonucleic acid
NGS: Next-generation sequencing
NS: Nasal swabs
OTU: Operational taxonomic unit
PCoA: Principal coordinates analysis
PERMANOVA: Permutational multivariate analysis of variance
QIIME: Quantitative Insights Into Microbial Ecology
TTA: Trans-tracheal aspiration

## References

[1] Tablerlet P, Coissac E, Pompanon F, Brochmann C, Willerslev E (2012). Towards next-generation biodiversity assessment using DNA metabarcoding. Mol Ecol.

[2] Segal LN, Rom WN, Weiden MD (2014). Lung microbiome for clinicians: new discoveries about bugs in healthy and diseased lungs. Ann Am Thorac Soc.

[3] Charlson ES, Bittinger K, Haas AR, Fitzgerald AS, Frank I, Yadav A (2011). Topographical continuity of bacterial populations in the healthy human respiratory tract. Am J Respir Crit Care Med.

[4] Dickson RP, Erb-Downward JR, Huffnagle GB (2014). Towards an ecology of the lung: new conceptual models of pulmonary microbiology and pneumonia pathogenesis. Lancet Respir. Med..

[5] Morris A, Beck JM, Schloss PD, Campbell TB, Crothers K, Curtis JL (2013). Comparison of the respiratory microbiome in healthy nonsmokers and smokers. Am J Respir Crit Care Med.

[6] Dickson RP, Martinez FJ, Huffnagle GB (2014). The role of the microbiome in exacerbations of chronic lung diseases. Lancet.

[7] Huffnagle GB, Dickson RP (2015). The bacterial microbiota in inflammatory lung diseases. Clin Immunol.

[8] Edwards TA (2010). Control methods for bovine respiratory disease for feedlot cattle. Vet Clin North Am - Food Anim Pract.

[9] Woolums AR. Lower respiratory tract disease. In: BP S, editor. Large Anim. Intern. Med. Fifth edition. St. Louis (MO): Mosby Elsevier; 2015. p. 583–617.

[10] Pardon B, De Bleecker K, Hostens M, Callens J, Dewulf J, Deprez P (2012). Longitudinal study on morbidity and mortality in white veal calves in Belgium. BMC Vet Res.

[11] Brscic M, Leruste H, Heutinck LFM, Bokkers EAM, Wolthuis-Fillerup M, Stockhofe N (2012). Prevalence of respiratory disorders in veal calves and potential risk factors. J Dairy Sci.

[12] Gay E, Barnouin J (2009). A nation-wide epidemiological study of acute bovine respiratory disease in France. Prev. Vet. Med..

[13] Assié S, Seegers H, Makoschey B, Désiré-Bousquié L, Bareille N (2009). Exposure to pathogens and incidence of respiratory disease in young bulls on their arrival at fattening operations in France. Vet. Rec..

[14] Stilwell G, Matos M, Carolino N, Lima MS. Effect of a quadrivalent vaccine against respiratory virus on the incidence of respiratory disease in weaned beef calves. Prev. Vet. Med. 2008; 10.1016/j.prevetmed.2008.02.002 PMC7127212 18378342

[15] Svensson C, Hultgren J, Oltenacu PA (2006). Morbidity in 3–7-month-old dairy calves in south-western Sweden, and risk factors for diarrhoea and respiratory disease. Prev Vet Med.

[16] Svensson C, Linder A, Olsson S-O (2006). Mortality in Swedish dairy calves and replacement heifers. J Dairy Sci.

[17] Nickell JS, White BJ (2010). Metaphylactic antimicrobial therapy for bovine respiratory disease in stocker and feedlot cattle. Vet. Clin. North Am. - Food Anim. Pract.

[18] Portis E, Lindeman C, Johansen L, Stoltman G (2012). A ten-year (2000-2009) study of antimicrobial susceptibility of bacteria that cause bovine respiratory disease complex—Mannheimia haemolytica, Pasteurella multocida, and Histophilus somni—in the United States and Canada. J Vet Diagnostic Investig.

[19] Marshall BM, Levy SB (2011). Food animals and antimicrobials: impacts on human health. Clin Microbiol Rev.

[20] Panciera RJ, Confer AW (2010). Pathogenesis and pathology of bovine pneumonia. Vet. Clin. North Am. - Food Anim. Pract.

[21] Taylor JD, Fulton RW, Lehenbauer TW, Step DL, Confer AW (2010). The epidemiology of bovine respiratory disease: what is the evidence for predisposing factors?. Can Vet J.

[22] Griffin D. Bovine pasteurellosis and other bacterial infections of the respiratory tract. Vet. Clin. North Am. - Food Anim. Pract 2010;26:57–71. 10.1016/j.cvfa.2009.10.010 20117542

[23] Angen Ø, Thomsen J, Larsen LE, Larsen J, Kokotovic B, Heegaard PMH (2009). Respiratory disease in calves: microbiological investigations on trans-tracheally aspirated bronchoalveolar fluid and acute phase protein response. Vet Microbiol.

[24] Allen JW, Viel L, Bateman KG, Rosendal S, Shewen PE, Physick-Sheard P (1991). The microbial flora of the respiratory tract in feedlot calves: associations between nasopharyngeal and bronchoalveolar lavage cultures. Can J Vet Res.

[25] Holman DB, Timsit E, Alexander TW, Checkley SL, Campbell JR, Chirino-Trejo M (2015). The nasopharyngeal microbiota of feedlot cattle. Sci Rep.

[26] Timsit E, Workentine M, Schryvers AB, Holman DB, van der Meer F, Alexander TW (2016). Evolution of the nasopharyngeal microbiota of beef cattle from weaning to 40 days after arrival at a feedlot. Vet Microbiol.

[27] Holman DB, McAllister TA, Topp E, Wright A-DG, Alexander TW (2015). The nasopharyngeal microbiota of feedlot cattle that develop bovine respiratory disease. Vet Microbiol.

[28] DB Holman, E Timsit, S Amat, D. Wade Abbott, AG. Buret and TW. Alexander. The nasopharyngeal microbiota of beef cattle before and after transport to a feedlot. BMC Microbiol 2017;17:1–12. 10.1186/s12866-017-0978-6 PMC5361731 28330466

[29] Timsit E, Workentine M, Crepieux T, Miller C, Regev-Shoshani G, Schaefer A (2017). Effects of nasal instillation of a nitric oxide-releasing solution or parenteral administration of tilmicosin on the nasopharyngeal microbiota of beef feedlot cattle at high-risk of developing respiratory tract disease. Res Vet Sci.

[30] Gaeta NC, Lima SF, Teixeira AG, Ganda EK, Oikonomou G, Gregory L, et al. Deciphering upper respiratory tract microbiota complexity in healthy calves and calves that develop respiratory disease using shotgun metagenomics. J Dairy Sci. 2017:1–14. 10.3168/jds.2016-11522 27988122

[31] Lima SF, Teixeira AGV, Higgins CH, Lima FS, Bicalho RC (2016). The upper respiratory tract microbiome and its potential role in bovine respiratory disease and otitis media. Sci Rep.

[32] Klindworth A, Pruesse E, Schweer T, Peplies J, Quast C, Horn M (2013). Evaluation of general 16S ribosomal RNA gene PCR primers for classical and next-generation sequencing-based diversity studies. Nucleic Acids Res.

[33] Caporaso JG, Kuczynski J, Stombaugh J, Bittinger K, Bushman FD, Costello EK (2010). QIIME allows analysis of high-throughput community sequencing data. Nat Methods.

[34] Edgar RC (2010). Search and clustering orders of magnitude faster than BLAST. Bioinformatics.

[35] Haas BJ, Gevers D, Earl AM, Feldgarden M, Ward DV, Giannoukos G (2011). Chimeric 16S rRNA sequence formation and detection in Sanger and 454-pyrosequenced PCR amplicons. Genome Res.

[36] Love MI, Huber W, Anders S (2014). Moderated estimation of fold change and dispersion for RNA-seq data with DESeq2. Genome Biol.

[37] Benjamini Y, Hochberg Y (1995). Controlling the false discovery rate: a practical and powerful approach to multiple testing. J R Stat Soc B.

[38] La Rosa PS, Brooks JP, Deych E, Boone EL, Edwards DJ, Wang Q, et al. Hypothesis testing and power calculations for taxonomic-based human microbiome data. White EP, editor. PLoS One 2012;7:e52078. 10.1371/journal.pone.0052078 PMC3527355 23284876

[39] Bosch AATM, Levin E, van Houten MA, Hasrat R, Kalkman G, Biesbroek G (2016). Development of upper respiratory tract microbiota in infancy is affected by mode of delivery. EBioMedicine.

[40] Biesbroek G, Bosch AATM, Wang X, Keijser BJF, Veenhoven RH, Sanders EA (2014). The impact of breastfeeding on nasopharyngeal microbial communities in infants. Am J Respir Crit Care Med.

[41] Stressmann FA, Rogers GB, Klem ER, Lilley AK, Donaldson SH, Daniels TW (2011). Analysis of the bacterial communities present in lungs of patients with cystic fibrosis from American and British centers. J Clin Microbiol.

[42] Salter SJ, Cox MJ, Turek EM, Calus ST, Cookson WO, Moffatt MF (2014). Reagent and laboratory contamination can critically impact sequence-based microbiome analyses. BMC Microbiol.

[43] Ericsson AC, Personett AR, Grobman ME, Rindt H, Reinero CR, Turnbaugh P (2016). Composition and predicted metabolic capacity of upper and lower airway microbiota of healthy dogs in relation to the fecal microbiota. PLoS One.

[44] Nikunen S, Härtel H, Orro T, Neuvonen E, Tanskanen R, Kivelä SL (2007). Association of bovine respiratory disease with clinical status and acute phase proteins in calves. Comp Immunol Microbiol Infect Dis.

[45] Thomas A, Ball H, Dizier I, Trolin A, Bell C, Mainil J (2002). Isolation of mycoplasma species from the lower respiratory tract of healthy cattle and cattle with respiratory disease in Belgium. Vet. Rec..

[46] Ayling RD, Bashiruddin SE, Nicholas RAJ (2004). Mycoplasma species and related organisms isolated from ruminants in Britain between 1990 and 2000. Vet Rec.

[47] Bertone I, Bellino C, Alborali GL, Cagnasso A, Cagnotti G, Dappiano E (2015). Clinical-pathological findings of otitis media and media-interna in calves and (clinical) evaluation of a standardized therapeutic protocol. BMC Vet Res BioMed Central.

[48] Allen JW, Viel L, Bateman KG, Rosendal S (1992). Changes in the bacterial flora of the upper and lower respiratory tracts and bronchoalveolar lavage differential cell counts in feedlot calves treated for respiratory diseases. Can J Vet Res.

[49] Angelos JA, Smith B (2015). Infectious bovine keratoconjunctivitis. Large animal internal medicine.

[50] Catry B, Boyen F, Baele M, Dewulf J, de Kruif A, Vaneechoutte M, et al. Recovery of Moraxella ovis from the bovine respiratory tract and differentiation of Moraxella species by tDNA-intergenic spacer PCR. Vet Microbiol. 2007;120:375–0. 10.1016/j.vetmic.2006.10.037 17141983

[51] Sakwinska O, Bastic Schmid V, Berger B, Bruttin A, Keitel K, Lepage M (2014). Nasopharyngeal microbiota in healthy children and pneumonia patients. J Clin Microbiol.

[52] Depner M, Ege MJ, Cox MJ, Dwyer S, Walker AW, Birzele LT, et al. Bacterial microbiota of the upper respiratory tract and childhood asthma. J Allergy Clin Immunol. 2016;139:826–34. 10.1016/j.jaci.2016.05.050 27576124

[53] Biesbroek G, Tsivtsivadze E, Sanders EAM, Montijn R, Veenhoven RH, Keijser BJF (2014). Early respiratory microbiota composition determines bacterial succession patterns and respiratory health in children. Am J Respir Crit Care Med.

[54] Laufer AS, Metlay JP, Gent JF, Fennie KP, Kong Y, Pettigrew MM (2011). Microbial communities of the upper respiratory tract and otitis media in children. MBio.

[55] Sturgeon A, Stull JW, Costa MC, Weese JS (2013). Metagenomic analysis of the canine oral cavity as revealed by high-throughput pyrosequencing of the 16S rRNA gene. Vet Microbiol.

[56] de Steenhuijsen Piters WAA, Sanders EAM, Bogaert D, Grice E, Segre J, Ding T (2015). The role of the local microbial ecosystem in respiratory health and disease. Philos Trans R Soc Lond Ser B Biol Sci.

[57] Erb-Downward JR, Thompson DL, Han MK, Freeman CM, McCloskey L, Schmidt LA (2011). Analysis of the lung microbiome in the “healthy” smoker and in COPD. PLoS One.

[58] Einarsson GG, Comer DM, McIlreavey L, Parkhill J, Ennis M, Tunney MM (2016). Community dynamics and the lower airway microbiota in stable chronic obstructive pulmonary disease, smokers and healthy non-smokers. Thorax.

[59] Lee SH, Sung JY, Yong D, Chun J, Kim SY, Song JH (2016). Characterization of microbiome in bronchoalveolar lavage fluid of patients with lung cancer comparing with benign mass like lesions. Lung Cancer.

[60] Bassis CM, Erb-Downward JR, Dickson RP, Freeman CM, Schmidt TM, Young VB (2015). Analysis of the upper respiratory tract microbiotas as the source of the lung and gastric microbiotas in healthy individuals. MBio.

[61] Glendinning L, Wright S, Pollock J, Tennant P, Collie D, McLachlan G. Variability of the sheep lung microbiota. Schloss PD, editor. Appl Environ Microbiol. 2016;82:3225–38. 10.1128/AEM.00540-16 PMC4959240 26994083

[62] Marsh RL, Kaestli M, Chang AB, Binks MJ, Pope CE, Hoffman LR (2016). The microbiota in bronchoalveolar lavage from young children with chronic lung disease includes taxa present in both the oropharynx and nasopharynx. Microbiome.

