# Supplementary figures and images for: Characterization of the upper and lower respiratory tract microbiota in Piedmontese calves
Source: Microbiome. 2017 Nov 21;5:152. doi: 10.1186/s40168-017-0372-5 (PMC5697440; doi:10.1186/s40168-017-0372-5)

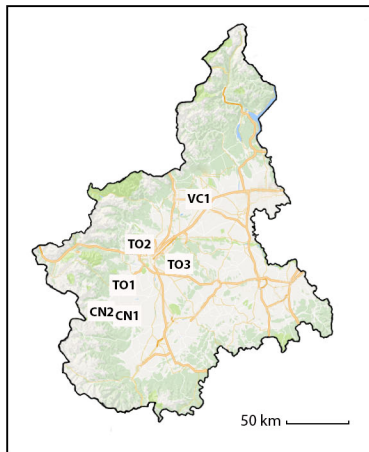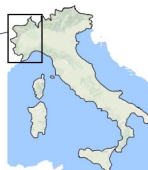

|            | Latitude      | Longitude         |
|------------|---------------|-------------------|
| <b>TO1</b> | 44.8059868    | 7.390839499999997 |
| <b>CN1</b> | 44.6483266    | 7.392150600000036 |
| <b>CN2</b> | 44.6777212999 | 7.279322200000024 |
| <b>VC1</b> | 45.3540681    | 8.0562228         |
| <b>TO2</b> | 45.0859687    | 7.539703000000031 |
| <b>TO3</b> | 44.96996      | 7.877819999999929 |

Supplement: Supplementary file 1 — Geographical distribution of the farms. Maps were adapted from Google Maps. (PDF 158 kb) [file 40168_2017_372_MOESM1_ESM.pdf]
