# Supplementary material for: Characterization of the upper and lower respiratory tract microbiota in Piedmontese calves
Source: Microbiome. 2017 Nov 21;5:152. doi: 10.1186/s40168-017-0372-5 (PMC5697440; doi:10.1186/s40168-017-0372-5)
Supplement: Supplementary file 2 — Relative abundance of genera identified in the nasal swab (NS) and the trans-tracheal aspiration (TTA) samples. Genera identified only in the NS (n = 11) or in the TTA (n = 17) samples are shown in bold or underlined, respectively. Table S2. Operational taxonomic units (OTUs) identified at the species level in the nasal swab (NS) and trans-tracheal aspiration (TTA) samples. (DOCX 44 kb) [file 40168_2017_372_MOESM2_ESM.docx]

ADDITIONAL FILES 2

**Table S1***.* Relative abundance of genera identified in the nasal swab (NS) and the trans-tracheal
aspiration (TTA) samples. Data are reported as average relative abundance and standard error of the mean (SEM). Genera identified only in the NS (n = 11) or in the TTA (n = 17) samples are shown in bold or underlined, respectively.

|  | **TTA** | | **NS** | |
| --- | --- | --- | --- | --- |
|  | **Mean (%)** | **SEM (%)** | **Mean (%)** | **SEM (%)** |
| *Mycoplasma* | 72.9898 | 5.5536 | 35.0538 | 6.9365 |
| *Pasteurella* | 7.6046 | 3.7231 | 0.6007 | 0.4301 |
| *Mannheimia* | 0.7287 | 0.6186 | 1.0765 | 1.0642 |
| *Bacteroides* | 1.8195 | 1.8044 | 0.3423 | 0.0656 |
| *Ureaplasma* | 1.2664 | 0.6547 | 0.0867 | 0.0392 |
| *Prevotella* | 0.4990 | 0.4545 | 0.8666 | 0.2026 |
| *Helcococcus* | 0.3110 | 0.3089 | 0.0151 | 0.0064 |
| *Moraxella* | 0.3292 | 0.1628 | 5.9807 | 4.7562 |
| *Fusobacterium* | 0.2559 | 0.2431 | 0.0039 | 0.0031 |
| *Sphingomonas* | 0.1499 | 0.0827 | 2.5698 | 0.8526 |
| *Agrobacterium* | 0.0905 | 0.0302 | 0.5275 | 0.1772 |
| *Porphyromonas* | 0.1144 | 0.0998 | 0.0021 | 0.0013 |
| *Corynebacterium* | 0.0477 | 0.0211 | 1.6248 | 0.2345 |
| *Delftia* | 0.0920 | 0.0263 | 0.1956 | 0.0525 |
| *Parvimonas* | 0.0582 | 0.0552 | 0.0000 | 0.0000 |
| *Campylobacter* | 0.0508 | 0.0500 | 0.0090 | 0.0067 |
| *Pedobacter* | 0.0258 | 0.0180 | 0.3597 | 0.1445 |
| *Coprococcus* | 0.0279 | 0.0152 | 1.0294 | 0.2634 |
| *Methylobacterium* | 0.0276 | 0.0131 | 0.4836 | 0.1997 |
| *Propionibacterium* | 0.0203 | 0.0153 | 0.0373 | 0.0121 |
| *Acinetobacter* | 0.0285 | 0.0117 | 0.8845 | 0.1718 |
| *Sphingobium* | 0.0228 | 0.0109 | 0.2365 | 0.0950 |
| *Ruminobacter* | 0.0146 | 0.0101 | 0.7804 | 0.2367 |
| *Blautia* | 0.0206 | 0.0095 | 1.0208 | 0.2065 |
| *Chryseobacterium* | 0.0150 | 0.0104 | 0.2309 | 0.1003 |
| *Streptococcus* | 0.0390 | 0.0295 | 0.3811 | 0.1415 |
| *Hymenobacter* | 0.0111 | 0.0078 | 0.4047 | 0.1368 |
| *Anaerostipes* | 0.0097 | 0.0076 | 0.1285 | 0.0418 |
| *Ruminococcus* | 0.0108 | 0.0063 | 0.4633 | 0.0923 |
| *[Prevotella]* | 0.0085 | 0.0070 | 0.3363 | 0.0599 |
| *Clostridium* | 0.0107 | 0.0061 | 0.1969 | 0.0390 |
| *Rhodococcus* | 0.0086 | 0.0062 | 0.1323 | 0.0512 |
| *Peptoniphilus* | 0.0157 | 0.0149 | 0.0014 | 0.0014 |
| *Pseudomonas* | 0.0098 | 0.0054 | 0.4204 | 0.0938 |
| *Faecalibacterium* | 0.0074 | 0.0054 | 0.3684 | 0.0869 |
| *Anaerococcus* | 0.0062 | 0.0061 | 0.0042 | 0.0024 |
| *Peptostreptococcus* | 0.0113 | 0.0102 | 0.0000 | 0.0000 |
| *Actinobacillus* | 0.0162 | 0.0066 | 0.0215 | 0.0128 |
| *Acholeplasma* | 0.0064 | 0.0045 | 0.1548 | 0.0353 |
| *Staphylococcus* | 0.0151 | 0.0105 | 0.2336 | 0.0697 |
| *Facklamia* | 0.0070 | 0.0032 | 0.3283 | 0.0493 |
| *Dorea* | 0.0091 | 0.0034 | 0.6892 | 0.1850 |
| *Succinivibrio* | 0.0062 | 0.0029 | 0.7054 | 0.1908 |
| *Butyrivibrio* | 0.0052 | 0.0023 | 0.2820 | 0.0675 |
| *Jeotgalicoccus* | 0.0050 | 0.0030 | 0.2788 | 0.0460 |
| *Psychrobacter* | 0.0108 | 0.0052 | 1.6173 | 0.6592 |
| *Flavobacterium* | 0.0049 | 0.0028 | 0.0692 | 0.0257 |
| *Luteimonas* | 0.0028 | 0.0025 | 0.0606 | 0.0177 |
| *Phascolarctobacterium* | 0.0037 | 0.0016 | 0.1760 | 0.0453 |
| *Trueperella* | 0.0036 | 0.0026 | 0.0059 | 0.0029 |
| *Turicibacter* | 0.0038 | 0.0018 | 0.1945 | 0.0419 |
| *Spirosoma* | 0.0024 | 0.0024 | 0.0152 | 0.0081 |
| *Leptotrichia* | 0.0102 | 0.0084 | 0.0112 | 0.0105 |
| *Micrococcus* | 0.0029 | 0.0017 | 0.0156 | 0.0063 |
| *Arthrobacter* | 0.0102 | 0.0076 | 0.1914 | 0.0585 |
| *Devosia* | 0.0044 | 0.0028 | 0.0665 | 0.0202 |
| *Candidatus Endobugula* | 0.0019 | 0.0017 | 0.1359 | 0.0292 |
| *Proteiniclasticum* | 0.0025 | 0.0018 | 0.0694 | 0.0259 |
| *Treponema* | 0.0019 | 0.0016 | 0.1370 | 0.0720 |
| *Chlamydia* | 0.0036 | 0.0036 | 0.0023 | 0.0023 |
| *Haemophilus* | 0.0017 | 0.0015 | 0.0004 | 0.0004 |
| *Novosphingobium* | 0.0036 | 0.0022 | 0.0023 | 0.0009 |
| *CF231* | 0.0057 | 0.0032 | 0.2383 | 0.1010 |
| *Mycetocola* | 0.0080 | 0.0052 | 0.0624 | 0.0212 |
| *Enhydrobacter* | 0.0025 | 0.0011 | 0.2980 | 0.1702 |
| *Rathayibacter* | 0.0017 | 0.0012 | 0.0448 | 0.0217 |
| *Arcobacter* | 0.0039 | 0.0028 | 0.0159 | 0.0063 |
| *Lactobacillus* | 0.0053 | 0.0039 | 0.1250 | 0.0489 |
| *Bacillus* | 0.0042 | 0.0015 | 0.0209 | 0.0074 |
| *Fibrobacter* | 0.0030 | 0.0020 | 0.0143 | 0.0069 |
| *Sanguibacter* | 0.0014 | 0.0013 | 0.0443 | 0.0153 |
| *Stenotrophomonas* | 0.0013 | 0.0013 | 0.0908 | 0.0418 |
| *Finegoldia* | 0.0013 | 0.0013 | 0.0000 | 0.0000 |
| *Halomonas* | 0.0012 | 0.0012 | 0.0318 | 0.0081 |
| *Anaerovibrio* | 0.0017 | 0.0012 | 0.1349 | 0.0280 |
| *Cloacibacterium* | 0.0036 | 0.0020 | 0.0004 | 0.0004 |
| *5-7N15* | 0.0024 | 0.0015 | 0.1448 | 0.0557 |
| *Planomicrobium* | 0.0012 | 0.0008 | 0.0767 | 0.0212 |
| *Paracoccus* | 0.0036 | 0.0026 | 0.0721 | 0.0168 |
| *Rothia* | 0.0011 | 0.0011 | 0.0023 | 0.0017 |
| *Dyadobacter* | 0.0027 | 0.0014 | 0.0330 | 0.0125 |
| *[Ruminococcus]* | 0.0010 | 0.0010 | 0.0686 | 0.0147 |
| *Granulicatella* | 0.0013 | 0.0009 | 0.0000 | 0.0000 |
| *Sutterella* | 0.0037 | 0.0026 | 0.1628 | 0.0501 |
| *Yaniella* | 0.0011 | 0.0008 | 0.0287 | 0.0079 |
| *Clavibacter* | 0.0010 | 0.0009 | 0.0384 | 0.0176 |
| *Dietzia* | 0.0055 | 0.0030 | 0.0805 | 0.0199 |
| *Janthinobacterium* | 0.0011 | 0.0009 | 0.0112 | 0.0035 |
| *Parabacteroides* | 0.0010 | 0.0007 | 0.0598 | 0.0162 |
| *Selenomonas* | 0.0010 | 0.0010 | 0.0003 | 0.0003 |
| *Aggregatibacter* | 0.0018 | 0.0011 | 1.2588 | 1.0055 |
| *Myroides* | 0.0023 | 0.0015 | 0.1584 | 0.0569 |
| *Aerococcus* | 0.0026 | 0.0014 | 0.1011 | 0.0256 |
| *Microbacterium* | 0.0009 | 0.0007 | 0.0150 | 0.0046 |
| *Cellulomonas* | 0.0010 | 0.0006 | 0.0065 | 0.0042 |
| *Methanobrevibacter* | 0.0009 | 0.0005 | 0.0390 | 0.0140 |
| *Leucobacter* | 0.0010 | 0.0006 | 0.0124 | 0.0056 |
| *Succiniclasticum* | 0.0011 | 0.0008 | 0.0031 | 0.0017 |
| *Cardiobacterium* | 0.0006 | 0.0006 | 0.0000 | 0.0000 |
| *Ochrobactrum* | 0.0007 | 0.0005 | 0.0090 | 0.0072 |
| *Solibacillus* | 0.0007 | 0.0005 | 0.0940 | 0.0255 |
| *Bulleidia* | 0.0006 | 0.0005 | 0.0730 | 0.0360 |
| *Deinococcus* | 0.0051 | 0.0040 | 0.0125 | 0.0054 |
| *[Eubacterium]* | 0.0005 | 0.0004 | 0.0448 | 0.0150 |
| *Brevundimonas* | 0.0009 | 0.0006 | 0.0000 | 0.0000 |
| *Enterococcus* | 0.0012 | 0.0009 | 0.1669 | 0.0567 |
| *Meiothermus* | 0.0005 | 0.0005 | 0.0000 | 0.0000 |
| *Neisseria* | 0.0012 | 0.0007 | 0.0245 | 0.0154 |
| *Rhizobium* | 0.0014 | 0.0008 | 0.0054 | 0.0031 |
| *Bifidobacterium* | 0.0007 | 0.0003 | 0.0538 | 0.0108 |
| *Chroococcidiopsis* | 0.0005 | 0.0005 | 0.0000 | 0.0000 |
| *Kineococcus* | 0.0005 | 0.0005 | 0.0378 | 0.0181 |
| *Sediminibacterium* | 0.0008 | 0.0004 | 0.0002 | 0.0002 |
| *Ornithobacterium* | 0.0004 | 0.0004 | 0.0119 | 0.0063 |
| *Pseudoxanthomonas* | 0.0004 | 0.0004 | 0.0000 | 0.0000 |
| *Rummeliibacillus* | 0.0008 | 0.0004 | 0.0454 | 0.0126 |
| *Brachybacterium* | 0.0025 | 0.0014 | 0.0653 | 0.0177 |
| *Erwinia* | 0.0012 | 0.0007 | 0.1595 | 0.0878 |
| *Wautersiella* | 0.0012 | 0.0012 | 0.0623 | 0.0203 |
| *Coprobacillus* | 0.0016 | 0.0013 | 0.0210 | 0.0078 |
| *Trichococcus* | 0.0005 | 0.0002 | 0.0863 | 0.0204 |
| *Veillonella* | 0.0005 | 0.0003 | 0.0123 | 0.0119 |
| *Cellvibrio* | 0.0019 | 0.0019 | 0.0381 | 0.0118 |
| *Comamonas* | 0.0003 | 0.0002 | 0.0112 | 0.0045 |
| *Erysipelothrix* | 0.0003 | 0.0002 | 0.0729 | 0.0181 |
| *Filifactor* | 0.0007 | 0.0007 | 0.0000 | 0.0000 |
| *Oscillospira* | 0.0006 | 0.0004 | 0.2663 | 0.0608 |
| *Salinicoccus* | 0.0003 | 0.0003 | 0.0053 | 0.0026 |
| *vadinCA11* | 0.0002 | 0.0002 | 0.0000 | 0.0000 |
| *BD2-13* | 0.0003 | 0.0002 | 0.0214 | 0.0119 |
| *Guggenheimella* | 0.0003 | 0.0002 | 0.0279 | 0.0106 |
| *Shuttleworthia* | 0.0003 | 0.0003 | 0.0137 | 0.0050 |
| *Akkermansia* | 0.0006 | 0.0006 | 0.0163 | 0.0074 |
| *Anaeroplasma* | 0.0003 | 0.0002 | 0.0459 | 0.0247 |
| *Kocuria* | 0.0007 | 0.0006 | 0.0090 | 0.0045 |
| *Rhodobacter* | 0.0015 | 0.0013 | 0.0355 | 0.0197 |
| *Roseburia* | 0.0018 | 0.0017 | 0.0316 | 0.0142 |
| *Streptomyces* | 0.0002 | 0.0002 | 0.0321 | 0.0116 |
| *Tissierella* | 0.0004 | 0.0004 | 0.0130 | 0.0056 |
| *Actinomyces* | 0.0001 | 0.0001 | 0.0007 | 0.0007 |
| *Aeromicrobium* | 0.0001 | 0.0001 | 0.0275 | 0.0118 |
| *Cryocola* | 0.0001 | 0.0001 | 0.0024 | 0.0014 |
| *Morganella* | 0.0002 | 0.0002 | 0.0003 | 0.0003 |
| *Mycobacterium* | 0.0002 | 0.0001 | 0.0036 | 0.0015 |
| *p-75-a5* | 0.0002 | 0.0002 | 0.0043 | 0.0018 |
| *Sphingobacterium* | 0.0002 | 0.0002 | 0.0714 | 0.0280 |
| *Achromobacter* | 0.0001 | 0.0001 | 0.0000 | 0.0000 |
| *Agrococcus* | 0.0001 | 0.0001 | 0.0034 | 0.0015 |
| *Bibersteinia* | 0.0002 | 0.0002 | 0.0035 | 0.0030 |
| *Capnocytophaga* | 0.0001 | 0.0001 | 0.0000 | 0.0000 |
| *Luteococcus* | 0.0001 | 0.0001 | 0.0010 | 0.0010 |
| *Mesorhizobium* | 0.0002 | 0.0002 | 0.0000 | 0.0000 |
| *Natronobacillus* | 0.0001 | 0.0001 | 0.0098 | 0.0049 |
| *Nevskia* | 0.0002 | 0.0002 | 0.0003 | 0.0003 |
| *Ralstonia* | 0.0001 | 0.0001 | 0.0032 | 0.0031 |
| *RFN20* | 0.0001 | 0.0001 | 0.0190 | 0.0096 |
| *Rhodanobacter* | 0.0002 | 0.0002 | 0.0000 | 0.0000 |
| *Rhodoplanes* | 0.0002 | 0.0002 | 0.0023 | 0.0023 |
| *Saccharopolyspora* | 0.0001 | 0.0001 | 0.0487 | 0.0246 |
| *Tepidimonas* | 0.0002 | 0.0002 | 0.0000 | 0.0000 |
| *Tindallia* | 0.0001 | 0.0001 | 0.0011 | 0.0008 |
| *Varibaculum* | 0.0002 | 0.0002 | 0.0000 | 0.0000 |
| *YRC22* | 0.0002 | 0.0002 | 0.0027 | 0.0016 |
| *Brevibacterium* | 0.0003 | 0.0003 | 0.0249 | 0.0084 |
| *Cupriavidus* | 0.0001 | 0.0001 | 0.0011 | 0.0011 |
| *Curtobacterium* | 0.0000 | 0.0000 | 0.0010 | 0.0007 |
| *Demequina* | 0.0000 | 0.0000 | 0.0153 | 0.0098 |
| *GW-34* | 0.0001 | 0.0001 | 0.0233 | 0.0078 |
| *Lactococcus* | 0.0013 | 0.0013 | 0.0013 | 0.0007 |
| *Lautropia* | 0.0000 | 0.0000 | 0.0013 | 0.0008 |
| *Leuconostoc* | 0.0013 | 0.0013 | 0.0054 | 0.0039 |
| *Marinobacter* | 0.0001 | 0.0001 | 0.0112 | 0.0051 |
| *Patulibacter* | 0.0001 | 0.0001 | 0.0043 | 0.0041 |
| *Peptococcus* | 0.0001 | 0.0001 | 0.0032 | 0.0012 |
| *Propionivibrio* | 0.0001 | 0.0001 | 0.0007 | 0.0007 |
| *Pseudoclavibacter* | 0.0000 | 0.0000 | 0.0336 | 0.0110 |
| *Rheinheimera* | 0.0001 | 0.0001 | 0.0064 | 0.0037 |
| *Rudanella* | 0.0001 | 0.0001 | 0.0002 | 0.0002 |
| *Sharpea* | 0.0000 | 0.0000 | 0.0160 | 0.0124 |
| *Sporosarcina* | 0.0001 | 0.0001 | 0.0093 | 0.0047 |
| *Thauera* | 0.0003 | 0.0003 | 0.0048 | 0.0032 |
| *Vagococcus* | 0.0003 | 0.0003 | 0.0060 | 0.0045 |
| ***24838*** | 0.0000 | 0.0000 | 0.0035 | 0.0035 |
| ***Acetobacter*** | 0.0000 | 0.0000 | 0.0013 | 0.0013 |
| ***Acetobacterium*** | 0.0000 | 0.0000 | 0.0025 | 0.0025 |
| ***Acidaminococcus*** | 0.0000 | 0.0000 | 0.0006 | 0.0006 |
| ***Acidovorax*** | 0.0000 | 0.0000 | 0.0096 | 0.0052 |
| ***Adlercreutzia*** | 0.0000 | 0.0000 | 0.0031 | 0.0019 |
| ***Aequorivita*** | 0.0000 | 0.0000 | 0.0165 | 0.0046 |
| ***Alcanivorax*** | 0.0000 | 0.0000 | 0.0020 | 0.0015 |
| ***Alkalibacter*** | 0.0000 | 0.0000 | 0.0016 | 0.0016 |
| ***Alkalibacterium*** | 0.0000 | 0.0000 | 0.0064 | 0.0027 |
| ***Alkanindiges*** | 0.0000 | 0.0000 | 0.0004 | 0.0004 |
| ***Anaerofilum*** | 0.0000 | 0.0000 | 0.0002 | 0.0002 |
| ***Anaerolinea*** | 0.0000 | 0.0000 | 0.0034 | 0.0034 |
| ***Anaerospora*** | 0.0000 | 0.0000 | 0.0194 | 0.0117 |
| ***Arsenicicoccus*** | 0.0000 | 0.0000 | 0.0019 | 0.0019 |
| ***Atopobium*** | 0.0000 | 0.0000 | 0.0052 | 0.0025 |
| ***B-42*** | 0.0000 | 0.0000 | 0.0116 | 0.0065 |
| ***Bdellovibrio*** | 0.0000 | 0.0000 | 0.0084 | 0.0039 |
| ***BF311*** | 0.0000 | 0.0000 | 0.0005 | 0.0005 |
| ***Brumimicrobium*** | 0.0000 | 0.0000 | 0.0247 | 0.0099 |
| ***Caldilinea*** | 0.0000 | 0.0000 | 0.0023 | 0.0023 |
| ***Candidatus Arthromitus*** | 0.0000 | 0.0000 | 0.0017 | 0.0017 |
| ***Candidatus Portiera*** | 0.0000 | 0.0000 | 0.0178 | 0.0078 |
| ***Carnobacterium*** | 0.0000 | 0.0000 | 0.0014 | 0.0009 |
| ***Catenibacterium*** | 0.0000 | 0.0000 | 0.0021 | 0.0021 |
| ***Chelonobacter*** | 0.0000 | 0.0000 | 0.0002 | 0.0002 |
| ***Chitinophaga*** | 0.0000 | 0.0000 | 0.0091 | 0.0091 |
| ***Collinsella*** | 0.0000 | 0.0000 | 0.0024 | 0.0015 |
| ***Cryomorpha*** | 0.0000 | 0.0000 | 0.0011 | 0.0011 |
| ***Cytophaga*** | 0.0000 | 0.0000 | 0.0023 | 0.0023 |
| ***Dermabacter*** | 0.0000 | 0.0000 | 0.0008 | 0.0005 |
| ***Desemzia*** | 0.0000 | 0.0000 | 0.0052 | 0.0038 |
| ***Desulfobulbus*** | 0.0000 | 0.0000 | 0.0027 | 0.0016 |
| ***Desulfovibrio*** | 0.0000 | 0.0000 | 0.0006 | 0.0006 |
| ***Dialister*** | 0.0000 | 0.0000 | 0.0002 | 0.0002 |
| ***Dokdonella*** | 0.0000 | 0.0000 | 0.0042 | 0.0042 |
| ***Dysgonomonas*** | 0.0000 | 0.0000 | 0.0027 | 0.0018 |
| ***Ellin506*** | 0.0000 | 0.0000 | 0.0016 | 0.0016 |
| ***Enterobacter*** | 0.0000 | 0.0000 | 0.0009 | 0.0009 |
| ***Epulopiscium*** | 0.0000 | 0.0000 | 0.0272 | 0.0205 |
| ***Euzebya*** | 0.0000 | 0.0000 | 0.0003 | 0.0003 |
| ***Flavisolibacter*** | 0.0000 | 0.0000 | 0.0013 | 0.0007 |
| ***Flectobacillus*** | 0.0000 | 0.0000 | 0.0019 | 0.0019 |
| ***Fluviicola*** | 0.0000 | 0.0000 | 0.0123 | 0.0052 |
| ***Friedmanniella*** | 0.0000 | 0.0000 | 0.0014 | 0.0013 |
| ***Gallicola*** | 0.0000 | 0.0000 | 0.0148 | 0.0105 |
| ***Gelidibacter*** | 0.0000 | 0.0000 | 0.0151 | 0.0086 |
| ***Georgenia*** | 0.0000 | 0.0000 | 0.0086 | 0.0030 |
| ***Gordonia*** | 0.0000 | 0.0000 | 0.0025 | 0.0017 |
| ***HTCC*** | 0.0000 | 0.0000 | 0.0009 | 0.0009 |
| ***Hydrogenophaga*** | 0.0000 | 0.0000 | 0.0015 | 0.0008 |
| ***Hylemonella*** | 0.0000 | 0.0000 | 0.0021 | 0.0021 |
| ***Jonesia*** | 0.0000 | 0.0000 | 0.0041 | 0.0025 |
| ***Kaistobacter*** | 0.0000 | 0.0000 | 0.0033 | 0.0022 |
| ***Klebsiella*** | 0.0000 | 0.0000 | 0.0014 | 0.0014 |
| ***Kurthia*** | 0.0000 | 0.0000 | 0.0041 | 0.0027 |
| ***Labrys*** | 0.0000 | 0.0000 | 0.0030 | 0.0030 |
| ***Lachnobacterium*** | 0.0000 | 0.0000 | 0.0402 | 0.0125 |
| ***Lachnospira*** | 0.0000 | 0.0000 | 0.0106 | 0.0062 |
| ***Leadbetterella*** | 0.0000 | 0.0000 | 0.0011 | 0.0011 |
| ***Legionella*** | 0.0000 | 0.0000 | 0.0006 | 0.0006 |
| ***Luteibacter*** | 0.0000 | 0.0000 | 0.0033 | 0.0026 |
| ***Luteolibacter*** | 0.0000 | 0.0000 | 0.0018 | 0.0018 |
| ***Lysinibacillus*** | 0.0000 | 0.0000 | 0.0033 | 0.0017 |
| ***Marinilactibacillus*** | 0.0000 | 0.0000 | 0.0034 | 0.0034 |
| ***Marinococcus*** | 0.0000 | 0.0000 | 0.0015 | 0.0013 |
| ***Megamonas*** | 0.0000 | 0.0000 | 0.0008 | 0.0006 |
| ***Megasphaera*** | 0.0000 | 0.0000 | 0.0051 | 0.0029 |
| ***Methanosphaera*** | 0.0000 | 0.0000 | 0.0045 | 0.0023 |
| ***Methylibium*** | 0.0000 | 0.0000 | 0.0018 | 0.0018 |
| ***Methylophaga*** | 0.0000 | 0.0000 | 0.0119 | 0.0077 |
| ***Methylotenera*** | 0.0000 | 0.0000 | 0.0018 | 0.0018 |
| ***Microbispora*** | 0.0000 | 0.0000 | 0.0023 | 0.0016 |
| ***Mitsuokella*** | 0.0000 | 0.0000 | 0.0008 | 0.0008 |
| ***Mogibacterium*** | 0.0000 | 0.0000 | 0.0167 | 0.0050 |
| ***Moryella*** | 0.0000 | 0.0000 | 0.0030 | 0.0030 |
| ***ND137*** | 0.0000 | 0.0000 | 0.0009 | 0.0009 |
| ***Nesterenkonia*** | 0.0000 | 0.0000 | 0.0086 | 0.0046 |
| ***Niigata-25*** | 0.0000 | 0.0000 | 0.0006 | 0.0006 |
| ***Nitratireductor*** | 0.0000 | 0.0000 | 0.0021 | 0.0021 |
| ***Nocardioides*** | 0.0000 | 0.0000 | 0.0065 | 0.0044 |
| ***Nocardiopsis*** | 0.0000 | 0.0000 | 0.0024 | 0.0016 |
| ***Odoribacter*** | 0.0000 | 0.0000 | 0.0071 | 0.0042 |
| ***Oleibacter*** | 0.0000 | 0.0000 | 0.0031 | 0.0021 |
| ***Oligella*** | 0.0000 | 0.0000 | 0.0515 | 0.0197 |
| ***Olivibacter*** | 0.0000 | 0.0000 | 0.0066 | 0.0046 |
| ***Paenibacillus*** | 0.0000 | 0.0000 | 0.0107 | 0.0037 |
| ***Paludibacter*** | 0.0000 | 0.0000 | 0.0163 | 0.0057 |
| ***Pantoea*** | 0.0000 | 0.0000 | 0.0002 | 0.0002 |
| ***Paraprevotella*** | 0.0000 | 0.0000 | 0.0002 | 0.0002 |
| ***ph2*** | 0.0000 | 0.0000 | 0.0003 | 0.0003 |
| ***Phycicoccus*** | 0.0000 | 0.0000 | 0.0007 | 0.0005 |
| ***Phyllobacterium*** | 0.0000 | 0.0000 | 0.0009 | 0.0009 |
| ***Pigmentiphaga*** | 0.0000 | 0.0000 | 0.0054 | 0.0035 |
| ***Planctomyces*** | 0.0000 | 0.0000 | 0.0022 | 0.0016 |
| ***Prauserella*** | 0.0000 | 0.0000 | 0.0230 | 0.0116 |
| ***Propionicimonas*** | 0.0000 | 0.0000 | 0.0227 | 0.0085 |
| ***Pseudidiomarina*** | 0.0000 | 0.0000 | 0.0038 | 0.0017 |
| ***Pseudoalteromonas*** | 0.0000 | 0.0000 | 0.0006 | 0.0004 |
| ***Pseudonocardia*** | 0.0000 | 0.0000 | 0.0002 | 0.0002 |
| ***Pseudoramibacter*** | 0.0000 | 0.0000 | 0.0034 | 0.0027 |
| ***Pyramidobacter*** | 0.0000 | 0.0000 | 0.0022 | 0.0016 |
| ***rc4-4*** | 0.0000 | 0.0000 | 0.0139 | 0.0062 |
| ***Saccharomonospora*** | 0.0000 | 0.0000 | 0.0003 | 0.0003 |
| ***Salana*** | 0.0000 | 0.0000 | 0.0020 | 0.0012 |
| ***Sedimentibacter*** | 0.0000 | 0.0000 | 0.0172 | 0.0061 |
| ***Skermanella*** | 0.0000 | 0.0000 | 0.0009 | 0.0009 |
| ***SMB53*** | 0.0000 | 0.0000 | 0.0012 | 0.0010 |
| ***Sphaerochaeta*** | 0.0000 | 0.0000 | 0.0044 | 0.0032 |
| ***Terracoccus*** | 0.0000 | 0.0000 | 0.0022 | 0.0018 |
| ***Tessaracoccus*** | 0.0000 | 0.0000 | 0.0214 | 0.0056 |
| ***Variovorax*** | 0.0000 | 0.0000 | 0.0001 | 0.0001 |
| ***Vibrio*** | 0.0000 | 0.0000 | 0.0101 | 0.0057 |
| ***Vitreoscilla*** | 0.0000 | 0.0000 | 0.0014 | 0.0014 |
| ***Vogesella*** | 0.0000 | 0.0000 | 0.0025 | 0.0025 |
| ***W22*** | 0.0000 | 0.0000 | 0.0015 | 0.0011 |
| ***Weeksella*** | 0.0000 | 0.0000 | 0.0177 | 0.0088 |
| ***Weissella*** | 0.0000 | 0.0000 | 0.0014 | 0.0014 |
| ***Williamsia*** | 0.0000 | 0.0000 | 0.0075 | 0.0050 |
| ***Xanthobacter*** | 0.0000 | 0.0000 | 0.0076 | 0.0076 |
| ***Xylanimicrobium*** | 0.0000 | 0.0000 | 0.0084 | 0.0033 |
| ***Zhouia*** | 0.0000 | 0.0000 | 0.0024 | 0.0016 |
| ***Zoogloea*** | 0.0000 | 0.0000 | 0.0008 | 0.0008 |

Table S2. Operational taxonomic units (OTUs) identified at the species level in the nasal swab (NS)
and trans-tracheal aspiration (TTA) samples. Data are reported as average relative abundance and standard error of the mean (SEM).

|  | **TTA** | | **NS** | |
| --- | --- | --- | --- | --- |
|  | **Mean (%)** | **SEM (%)** | **Mean (%)** | **SEM (%)** |
| *Pasteurella multocida* | 7.6043 | 3.7230 | 0.6006 | 0.4301 |
| *Porphyromonas endodontalis* | 0.1004 | 0.1002 | 0.0000 | 0.0000 |
| *Propionibacterium acnes* | 0.0203 | 0.0153 | 0.0373 | 0.0121 |
| *Methylobacterium adhaesivum* | 0.0188 | 0.0128 | 0.3689 | 0.1526 |
| *Prevotella copri* | 0.0157 | 0.0114 | 0.4865 | 0.1269 |
| *Acinetobacter lwoffii* | 0.0132 | 0.0051 | 0.4644 | 0.0917 |
| *Rhodococcus fascians* | 0.0078 | 0.0057 | 0.1203 | 0.0485 |
| *Psychrobacter sanguinis* | 0.0077 | 0.0031 | 1.5771 | 0.6589 |
| *Faecalibacterium prausnitzii* | 0.0074 | 0.0054 | 0.3684 | 0.0869 |
| *Staphylococcus equorum* | 0.0063 | 0.0051 | 0.0443 | 0.0128 |
| *Actinobacillus capsulatus* | 0.0063 | 0.0027 | 0.0007 | 0.0007 |
| *Staphylococcus sciuri* | 0.0054 | 0.0051 | 0.0641 | 0.0286 |
| *Jeotgalicoccus psychrophilus* | 0.0048 | 0.0029 | 0.2165 | 0.0408 |
| *Blautia producta* | 0.0047 | 0.0028 | 0.2376 | 0.0563 |
| *Bacteroides eggerthii* | 0.0047 | 0.0038 | 0.0000 | 0.0000 |
| *Arcobacter cryaerophilus* | 0.0039 | 0.0028 | 0.0104 | 0.0041 |
| *Fibrobacter succinogenes* | 0.0030 | 0.0020 | 0.0143 | 0.0069 |
| *Clostridium neonatale* | 0.0028 | 0.0020 | 0.0107 | 0.0042 |
| *Brachybacterium conglomeratum* | 0.0025 | 0.0014 | 0.0636 | 0.0175 |
| *Sphingomonas yabuuchiae* | 0.0024 | 0.0014 | 0.0029 | 0.0020 |
| *Sphingomonas wittichii* | 0.0024 | 0.0016 | 0.0325 | 0.0184 |
| *Pedobacter cryoconitis* | 0.0021 | 0.0016 | 0.0464 | 0.0296 |
| *Novosphingobium capsulatum* | 0.0019 | 0.0015 | 0.0000 | 0.0000 |
| *Pseudomonas fragi* | 0.0017 | 0.0013 | 0.0012 | 0.0007 |
| *Rathayibacter caricis* | 0.0017 | 0.0012 | 0.0448 | 0.0217 |
| *Prevotella stercorea* | 0.0016 | 0.0010 | 0.1500 | 0.0332 |
| *Acinetobacter johnsonii* | 0.0016 | 0.0013 | 0.0307 | 0.0114 |
| *Haemophilus parainfluenzae* | 0.0015 | 0.0015 | 0.0004 | 0.0004 |
| *Rhizobium leguminosarum* | 0.0014 | 0.0008 | 0.0054 | 0.0031 |
| *Pseudomonas viridiflava* | 0.0013 | 0.0009 | 0.0909 | 0.0394 |
| *Rothia dentocariosa* | 0.0011 | 0.0011 | 0.0016 | 0.0016 |
| *Ruminococcus bromii* | 0.0010 | 0.0010 | 0.0010 | 0.0009 |
| *Sphingomonas echinoides* | 0.0010 | 0.0009 | 0.0192 | 0.0086 |
| *Brevundimonas diminuta* | 0.0009 | 0.0006 | 0.0000 | 0.0000 |
| *Paracoccus marcusii* | 0.0008 | 0.0008 | 0.0114 | 0.0053 |
| *Myroides odoratimimus* | 0.0008 | 0.0007 | 0.1118 | 0.0371 |
| *Kocuria rhizophila* | 0.0007 | 0.0006 | 0.0090 | 0.0045 |
| *Pseudomonas stutzeri* | 0.0005 | 0.0005 | 0.0060 | 0.0043 |
| *[Eubacterium] biforme* | 0.0005 | 0.0004 | 0.0316 | 0.0099 |
| *Veillonella dispar* | 0.0005 | 0.0003 | 0.0123 | 0.0119 |
| *Acholeplasma Laidlawii* | 0.0005 | 0.0004 | 0.0326 | 0.0106 |
| *Bacillus Cereus* | 0.0004 | 0.0002 | 0.0021 | 0.0007 |
| *Bacteroides coprophilus* | 0.0003 | 0.0002 | 0.0326 | 0.0078 |
| *Lactobacillus Brevis* | 0.0003 | 0.0003 | 0.0047 | 0.0028 |
| *Acinetobacter Schindleri* | 0.0003 | 0.0003 | 0.0005 | 0.0005 |
| *Corynebacterium Variabile* | 0.0003 | 0.0003 | 0.0141 | 0.0079 |
| *Morganella Morganii* | 0.0002 | 0.0002 | 0.0003 | 0.0003 |
| *Bacteroides Barnesiae* | 0.0002 | 0.0002 | 0.0000 | 0.0000 |
| *Bifidobacterium pseudolongum* | 0.0001 | 0.0001 | 0.0235 | 0.0052 |
| *Bulleidia p-1630-c5* | 0.0001 | 0.0001 | 0.0511 | 0.0341 |
| *Prevotella intermedia* | 0.0001 | 0.0001 | 0.0000 | 0.0000 |
| *Bacillus flexus* | 0.0001 | 0.0001 | 0.0000 | 0.0000 |
| *Roseburia faecis* | 0.0001 | 0.0001 | 0.0008 | 0.0004 |
| *Pseudoclavibacter bifida* | 0.0000 | 0.0000 | 0.0336 | 0.0110 |
| *Sharpea p-3329-23G2* | 0.0000 | 0.0000 | 0.0147 | 0.0117 |
| *[Eubacterium] cylindroides* | 0.0000 | 0.0000 | 0.0034 | 0.0024 |
| *[Eubacterium] dolichum* | 0.0000 | 0.0000 | 0.0097 | 0.0052 |
| *[Ruminococcus] gnavus* | 0.0000 | 0.0000 | 0.0006 | 0.0004 |
| *Agrococcus jenensis* | 0.0000 | 0.0000 | 0.0001 | 0.0001 |
| *Akkermansia muciniphila* | 0.0000 | 0.0000 | 0.0013 | 0.0013 |
| *Bacillus thermoamylovorans* | 0.0000 | 0.0000 | 0.0013 | 0.0013 |
| *Bacteroides fragilis* | 0.0000 | 0.0000 | 0.0004 | 0.0004 |
| *Bacteroides plebeius* | 0.0000 | 0.0000 | 0.0068 | 0.0026 |
| *Bacteroides uniformis* | 0.0000 | 0.0000 | 0.0028 | 0.0023 |
| *Bifidobacterium longum* | 0.0000 | 0.0000 | 0.0072 | 0.0037 |
| *Chelonobacter Taxon* | 0.0000 | 0.0000 | 0.0002 | 0.0002 |
| *Clostridium hiranonis* | 0.0000 | 0.0000 | 0.0001 | 0.0001 |
| *Clostridium perfringens* | 0.0000 | 0.0000 | 0.0008 | 0.0008 |
| *Collinsella aerofaciens* | 0.0000 | 0.0000 | 0.0023 | 0.0015 |
| *Coprococcus eutactus* | 0.0000 | 0.0000 | 0.0095 | 0.0054 |
| *Corynebacterium pilosum* | 0.0000 | 0.0000 | 0.0030 | 0.0017 |
| *Deinococcus aquatilis* | 0.0000 | 0.0000 | 0.0008 | 0.0008 |
| *Enterococcus cecorum* | 0.0000 | 0.0000 | 0.0046 | 0.0046 |
| *Flavobacterium succinicans* | 0.0000 | 0.0000 | 0.0016 | 0.0014 |
| *Janthinobacterium lividum* | 0.0000 | 0.0000 | 0.0002 | 0.0002 |
| *Lactobacillus agilis* | 0.0000 | 0.0000 | 0.0008 | 0.0008 |
| *Lactobacillus reuteri* | 0.0000 | 0.0000 | 0.0004 | 0.0004 |
| *Lactobacillus ruminis* | 0.0000 | 0.0000 | 0.0033 | 0.0024 |
| *Luteibacter rhizovicinus* | 0.0000 | 0.0000 | 0.0033 | 0.0026 |
| *Lysinibacillus boronitolerans* | 0.0000 | 0.0000 | 0.0033 | 0.0017 |
| *Marinilactibacillus psychrotolerans* | 0.0000 | 0.0000 | 0.0034 | 0.0034 |
| *Methylotenera mobilis* | 0.0000 | 0.0000 | 0.0018 | 0.0018 |
| *Microbispora rosea* | 0.0000 | 0.0000 | 0.0023 | 0.0016 |
| *Nocardioides plantarum* | 0.0000 | 0.0000 | 0.0010 | 0.0010 |
| *Nocardiopsis exhalans* | 0.0000 | 0.0000 | 0.0024 | 0.0016 |
| *Ochrobactrum intermedium* | 0.0000 | 0.0000 | 0.0006 | 0.0004 |
| *Pantoea agglomerans* | 0.0000 | 0.0000 | 0.0002 | 0.0002 |
| *Prauserella rugosa* | 0.0000 | 0.0000 | 0.0230 | 0.0116 |
| *Psychrobacter pacificensis* | 0.0000 | 0.0000 | 0.0024 | 0.0019 |
| *Rothia aeria* | 0.0000 | 0.0000 | 0.0008 | 0.0008 |
| *Ruminococcus flavefaciens* | 0.0000 | 0.0000 | 0.0085 | 0.0036 |
| *Saccharopolyspora hirsuta* | 0.0000 | 0.0000 | 0.0033 | 0.0020 |
| *Salana multivorans* | 0.0000 | 0.0000 | 0.0020 | 0.0012 |
| *Selenomonas ruminantium* | 0.0000 | 0.0000 | 0.0003 | 0.0003 |
| *Sphingobacterium faecium* | 0.0000 | 0.0000 | 0.0063 | 0.0022 |
| *Sphingobacterium mizutaii* | 0.0000 | 0.0000 | 0.0056 | 0.0031 |
| *Sphingobacterium multivorum* | 0.0000 | 0.0000 | 0.0030 | 0.0023 |
| *Streptococcus minor* | 0.0000 | 0.0000 | 0.0081 | 0.0049 |
| *Vibrio metschnikovii* | 0.0000 | 0.0000 | 0.0069 | 0.0041 |
| *Vibrio rumoiensis* | 0.0000 | 0.0000 | 0.0012 | 0.0012 |
