# Supplementary material for: Characterization of the upper and lower respiratory tract microbiota in Piedmontese calves
Source: Microbiome. 2017 Nov 21;5:152. doi: 10.1186/s40168-017-0372-5 (PMC5697440; doi:10.1186/s40168-017-0372-5)

ADDITIONAL FILE 3

Table S3. Alpha diversity metrics of each sample. The alpha diversity metrics include: Good's coverage, Chao1 and Observed species indices which estimate species richness; and Simpson and Shannon indices which estimate species evenness.

Sample_ID	Good's coverage	Chao1	Observed species	Simpson	Shannon
**4_TTA**	98.8%	97.6	56.0	0.73	2.33
**5_TTA**	99.5%	37.5	19.5	0.06	0.27
**3_TTA**	99.8%	20.9	14.0	0.53	1.24
**2_TTA**	99.5%	52.5	25.2	0.48	1.54
**21_TTA**	99.1%	88.2	40.6	0.40	1.35
**15_TTA**	98.5%	163.9	52.6	0.29	1.09
**16_TTA**	98.9%	107.6	36.8	0.58	1.67
**17_TTA**	99.4%	67.8	25.6	0.71	2.15
**10_TTA**	99.8%	12.9	7.7	0.03	0.15
**6_TTA**	96.0%	327.2	155.7	0.59	2.27
**7_TTA**	99.7%	28.9	12.2	0.25	0.68
**8_TTA**	99.1%	117.5	34.1	0.16	0.67
**13_TTA**	99.7%	24.2	13.3	0.19	0.62
**1_TTA**	99.4%	75.6	44.6	0.86	3.42
**14_TTA**	99.5%	50.1	22.1	0.55	1.34
**18_TTA**	99.3%	80.1	31.2	0.57	1.90
**20_TTA**	97.6%	200.7	99.2	0.64	2.17
**15_NS**	90.8%	788.1	540.8	0.98	7.53
**16_NS**	85.8%	1206.6	697.2	0.99	8.31
**10_NS**	92.0%	668.6	315.3	0.67	3.53
**11_NS**	91.1%	712.8	392.9	0.87	5.12
**6_NS**	90.0%	849.1	459.2	0.93	6.10
**7_NS**	90.1%	828.6	436.1	0.84	5.30
**8_NS**	93.1%	608.1	270.6	0.64	3.34
**12_NS**	92.7%	584.0	373.4	0.89	5.28
**13_NS**	91.3%	700.1	390.0	0.91	5.35
**18_NS**	91.5%	720.0	390.2	0.71	4.60
**20_NS**	98.5%	215.6	53.3	0.62	1.93

Fig. S1. Rarefaction curves for each sample for the Good's coverage index.

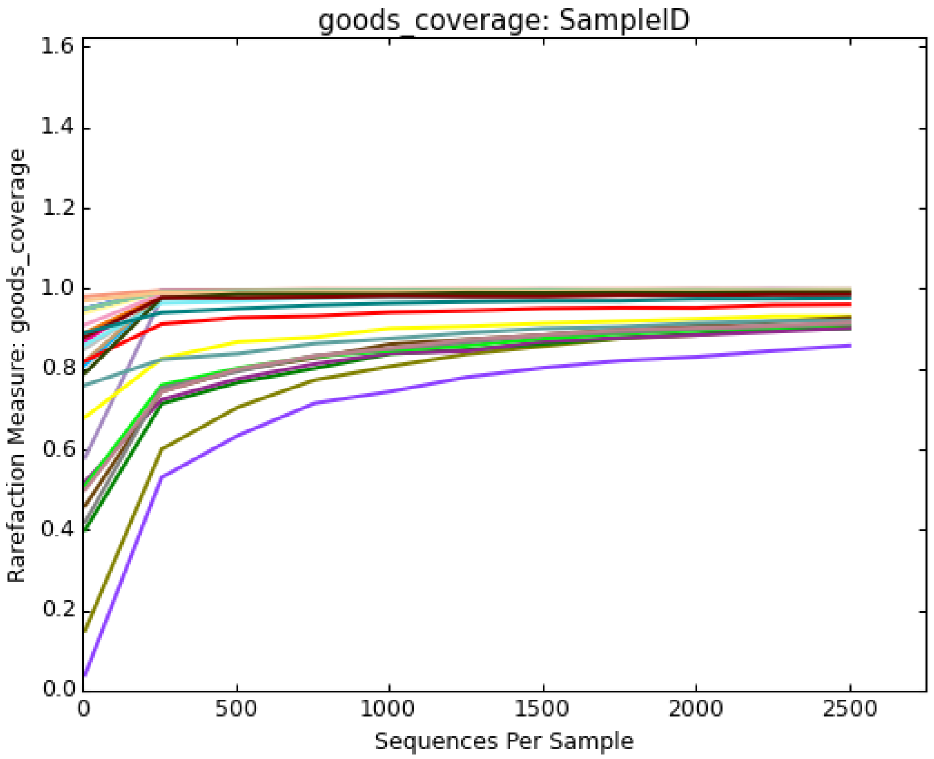

Fig. S2. Rarefaction curves for each sample for the Chao1 index.

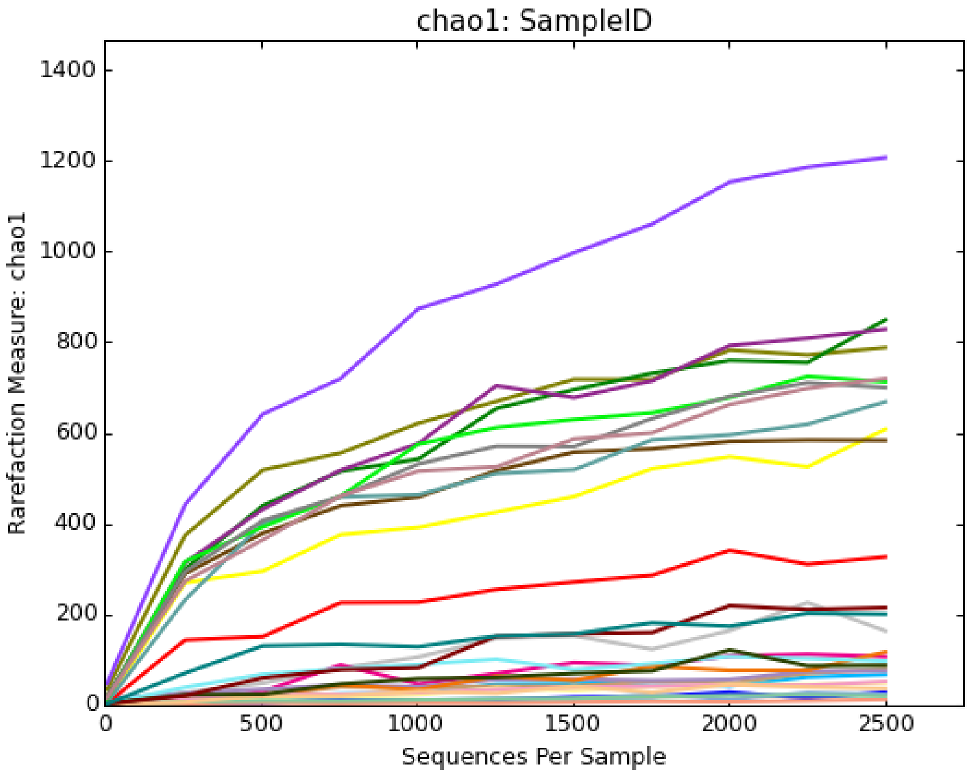

Fig. S3. Rarefaction curves for each sample for the Observed species index.

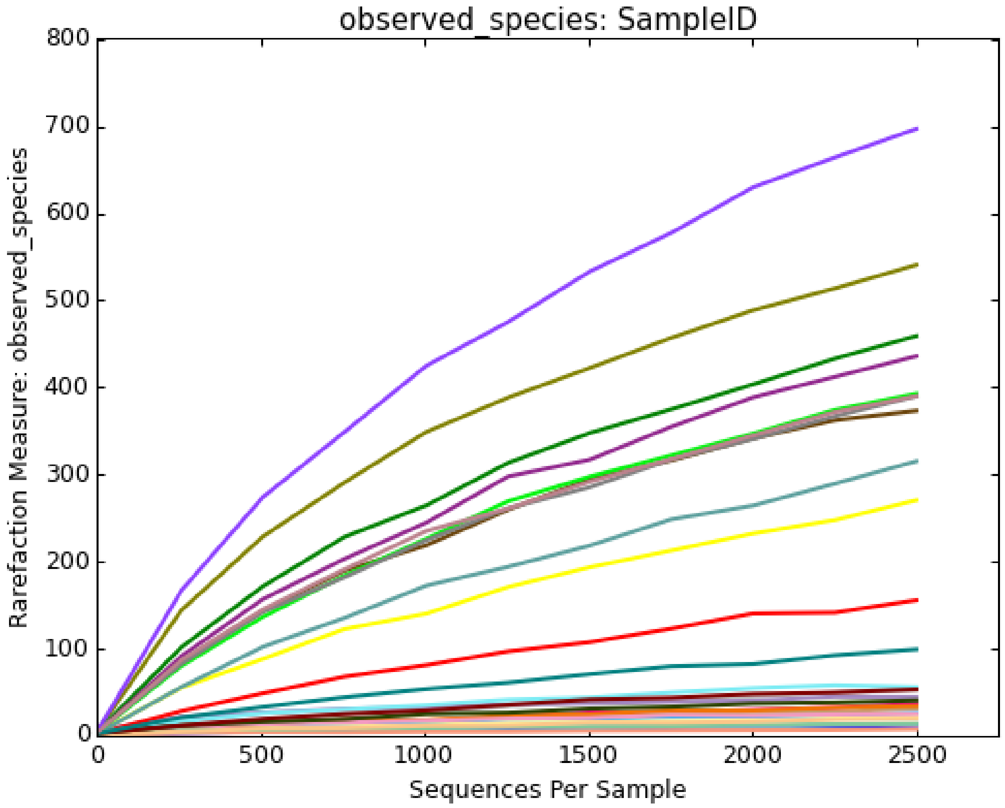

Fig. S4. Rarefaction curves for each sample for the Simpson index.

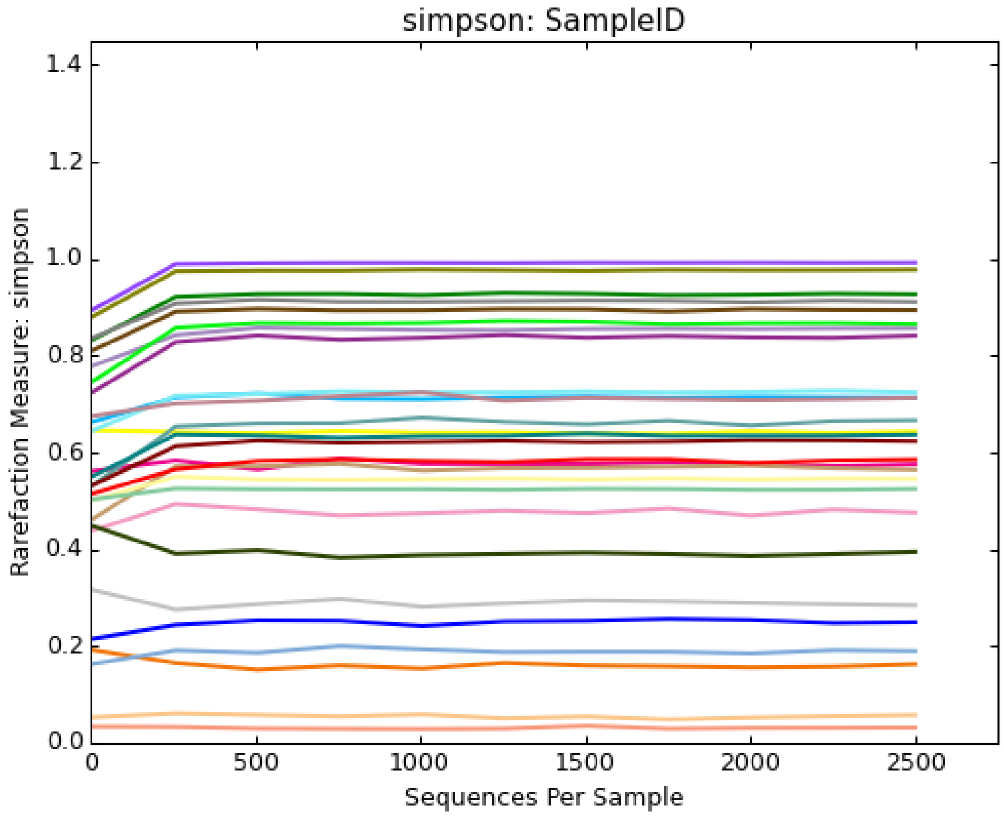

Fig. S5. Rarefaction curves for each sample for the Shannon index.

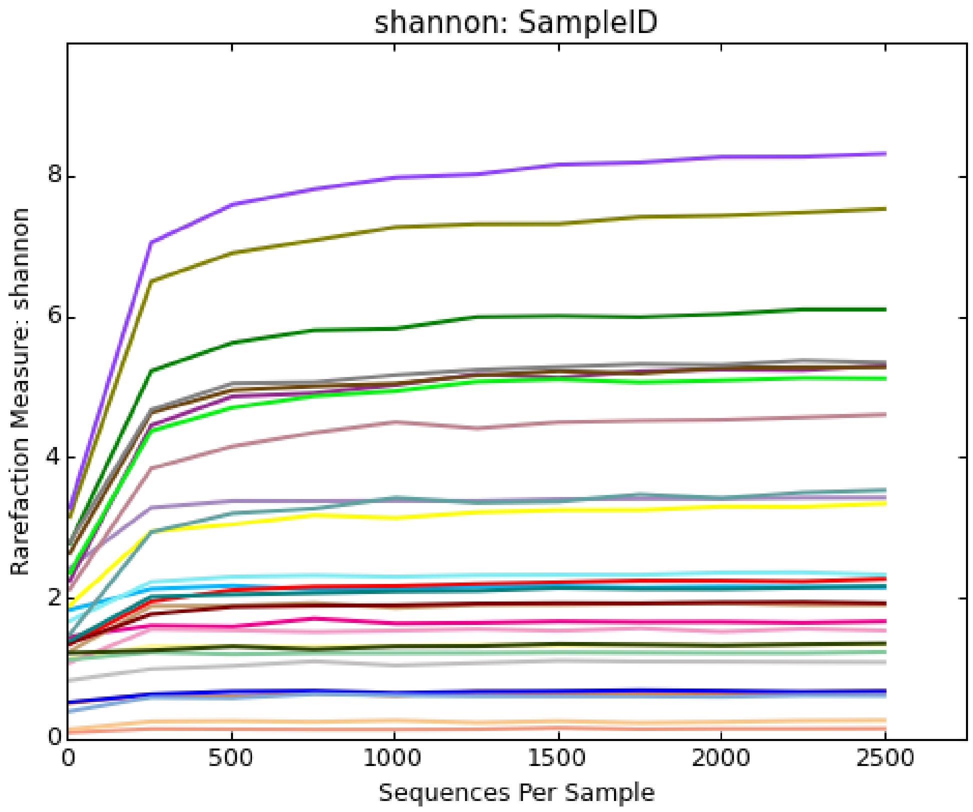

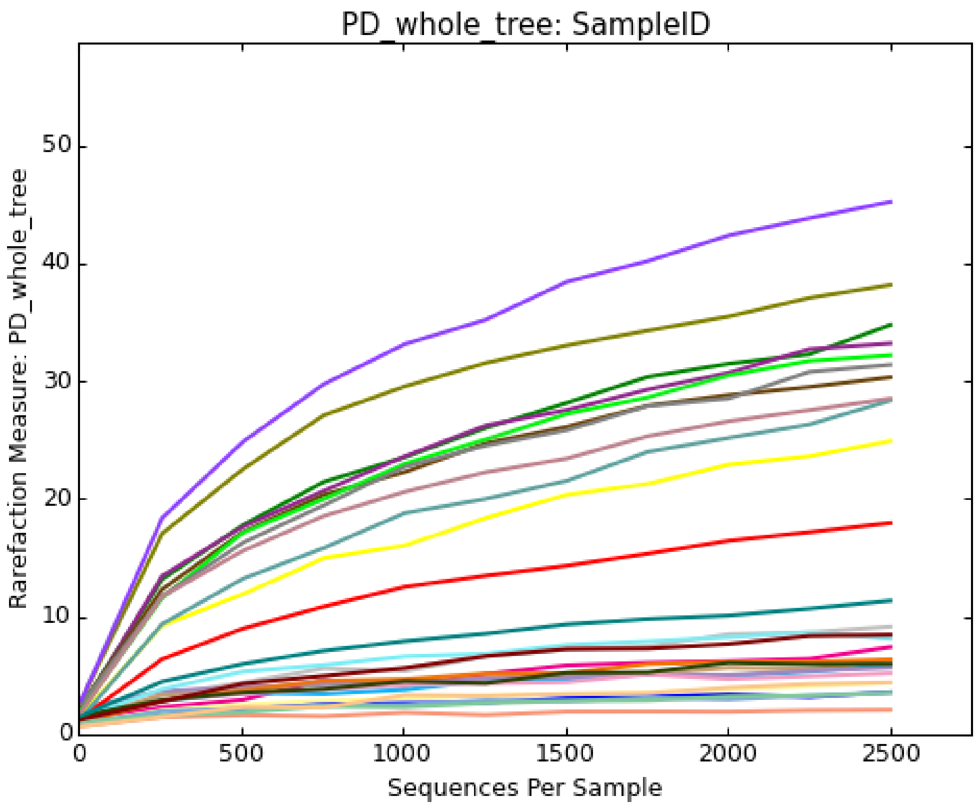

Supplement: Supplementary file 3 — Alpha diversity metrics per sample. The alpha diversity metrics include Good’s coverage, Chao1 and observed species indices which estimate species richness, and Simpson and Shannon indices which estimate species evenness. (PDF 827 kb) [file 40168_2017_372_MOESM3_ESM.pdf]
